# Impact of multiple infections on risk of incident dementia according to subjective cognitive decline status: a nationwide population-based cohort study

**DOI:** 10.3389/fnagi.2024.1410185

**Published:** 2024-09-02

**Authors:** Jung-Won Lee, Mina Kim, Hoseob Kim, Sunghwan Kim, Yoo Hyun Um, Sheng-Min Wang, Hyun Kook Lim, Chang Uk Lee, Dong Woo Kang

**Affiliations:** ^1^Department of Psychiatry, Seoul St. Mary’s Hospital, College of Medicine, The Catholic University of Korea, Seoul, Republic of Korea; ^2^Department of Data Science, Hanmi Pharm. Co., Ltd., Seoul, Republic of Korea; ^3^Department of Psychiatry, Yeouido St. Mary’s Hospital, College of Medicine, The Catholic University of Korea, Seoul, Republic of Korea; ^4^Department of Psychiatry, St. Vincent’s Hospital, College of Medicine, The Catholic University of Korea, Suwon, Republic of Korea; ^5^Research Institute, NEUROPHET Inc., Seoul, Republic of Korea

**Keywords:** dementia, subjective cognitive decline, infections, Alzheimer’s disease, cognitive status

## Abstract

**Background:**

The interrelation between infections, subjective cognitive decline (SCD), and dementia development is recognized, but not fully understood. This study explored the combined effect of specific infections and SCD on the risk of dementia.

**Objectives:**

To assess the influence of *Helicobacter pylori*, herpes simplex virus, varicella-zoster virus, and human papillomavirus on dementia risk in individuals with varying cognitive statuses, especially focusing on those with and without SCD.

**Methods:**

A cohort of 1,100,540 participants aged 66 years from the Korean National Health Insurance Service was divided into cognitively preserved (CP, *n* = 825,405) and SCD (*n* = 275,135) groups. This study analyzed the effects of single, dual, and triple infections on the risk of overall dementia, Alzheimer’s disease (AD), and vascular dementia (VaD) using incidence rates and hazard ratios.

**Results:**

The SCD group consistently showed a doubled risk of dementia, particularly AD, regardless of the number of infections. In the initial data, both the presence and number of infections, especially in the CP group, were associated with an increased dementia incidence and risk; however, this correlation disappeared after adjusting for covariates, hinting at a possible protective effect.

**Conclusion:**

Our findings emphasize that, while SCD is a steadfast risk factor for dementia, the role of infections is layered, subject to various influences, and requires more comprehensive exploration to fully understand their impact on dementia development.

## 1 Introduction

Dementia, defined as a decline in cognitive function, primarily in memory, leads to impaired daily activities and represents a significant global public health issue. Alzheimer’s disease (AD) is the most common form of dementia (Alzheimer’s Association, 2019). The Amyloid Cascade Hypothesis (ACH) is central to the understanding of AD, which suggests that amyloid-beta (Aβ) accumulation leads to neurofibrillary tangles, neurodegeneration, and cognitive decline (Barage and Sonawane, 2015). While the ACH has been central in understanding AD, the presence of Aβ in individuals without AD, and varying results from Aβ-targeted clinical trials, have led researchers to explore other contributing factors in the pathogenesis (Herrup, 2015; Shi et al., 2022). A significant focus has shifted towards the role of inflammation, tau pathology, and oxidative stress (Morris et al., 2014; Wang et al., 2016).

Among the other hypotheses proposed for the mechanism of dementia, the inflammation theory is of particular importance (Talwar et al., 2019). The inflammation theory of dementia suggests that chronic inflammation in the brain may contribute to neuronal damage and dysfunction in various ways, such as by exacerbating both Aβ and tau pathology, which can ultimately lead to cognitive decline and the development of dementia (Kinney et al., 2018). Infection is one of the factors that causes chronic inflammation, and it is believed that infections caused by pathogens such as viruses, bacteria, and fungi can contribute to the onset of dementia through various mechanisms, such as increasing tau phosphorylation or releasing inflammatory molecules such as cytokines in the brain (McManus and Heneka, 2017). The link between infections and dementia, although not fully established, is supported by evidence. *Helicobacter pylori* (*H. pylori*), herpes simplex virus (HSV), varicella-zoster virus (VZV), and human papillomavirus (HPV) are associated with an increased risk of dementia. *H. pylori* may contribute to Aβ deposition and tau phosphorylation in AD patients (Shindler-Itskovitch et al., 2016; McManus and Heneka, 2017), HSV-1 to increased Aβ production (McManus and Heneka, 2017), VZV might activate latent HSV-1, increasing the risk of dementia and exacerbating Aβ-related pathologies (Bubak et al., 2020; Cairns et al., 2022), and HPV is associated with a higher risk of dementia, potentially through oxidative stress and cytokine elevation (Lin et al., 2020). While previous studies have focused on individual pathogens, recent hypotheses suggest that the total infectious burden may provide a more accurate explanation for the occurrence of AD (Gale et al., 2016). The interactions among multiple infections across various taxa seem to influence cognitive function (Vigasova et al., 2021). This understanding must be framed within the context of the inherent complexities of dementia research. In clinical research exploring the impact of infections on the development of dementia, it is imperative to consider that dementia is a multifaceted neurodegenerative disease. This complexity implies that the potential risk factors influencing disease progression may not be sufficiently controlled in many studies. Furthermore, the majority of research on infection and dementia is observational in nature (Sipilä et al., 2021), often leading to varied interpretations owing to differing intensities of infections across studies. Additionally, meta-analyses in this field have demonstrated inconsistencies in the results of individual studies, with many encompassing low-quality overall evidence (Shindler-Itskovitch et al., 2016; Warren-Gash et al., 2019; Muzambi et al., 2020). Therefore, caution must be exercised in the interpretation of these research findings, given the heterogeneous nature of the studies and the nuanced understanding required for dementia as a multidimensional condition.

Subjective cognitive decline (SCD) in preclinical AD is increasingly being recognized as a significant risk factor for future cognitive decline, mild cognitive impairment (MCI), and AD dementia (Jessen et al., 2014). SCD is characterized by a self-perceived worsening of cognitive capacity despite normal objective performance levels on cognitive tests. Research has shown that individuals with SCD have approximately twice the risk of developing dementia than those with normal cognition, with a notable percentage progressing to dementia or MCI over time (Mitchell et al., 2014; Slot et al., 2019; Jessen et al., 2020). The prevalence and implications of SCD highlight the need for a differentiated approach in dementia research, especially in understanding how external factors, such as infections, impact cognitive health. Since SCD represents a critical phase in which intervention might alter the trajectory toward dementia, examining the influence of infections in this group is particularly relevant.

This study aimed to delve deeper into the interplay between infections and dementia, focusing on how different infection types and infectious burden impact dementia onset in individuals with and without SCD. Our research utilized a nationwide, population-based cohort, distinguished by its extensive scale, which encompasses a diverse and comprehensive representation of the population. This approach allows for an in-depth exploration of: (1) the impact of SCD on dementia onset according to the infection categorized by the number of infectious agents; (2) the effect of infections on dementia development within cognitively preserved (CP) and SCD groups; and (3) the comparative influence of single, dual, and triple infections on dementia risk across these cognitive statuses. The vast scope of our cohort, covering the entire nation, provides a unique and robust dataset that enhances the reliability and generalizability of our findings. This will enable a more accurate understanding of the relationship between infections and dementia and offer unparalleled insights into the epidemiological trends and risk factors associated with dementia. Our approach, underpinned by comprehensive data analysis, seeks to provide new insights into the multifactorial nature of dementia, particularly focusing on the interactions between SCD, infections, and cognitive decline.

## 2 Materials and methods

### 2.1 Data source

The Korean National Health Insurance Service (KNHIS) is a mandatory public health insurance system that provides universal coverage to all residents in South Korea (Song, 2009). All Koreans aged 40 years or older are required by the KNHIS, to receive a compulsory health screening test every two years. The National Health Insurance Service-Health Screening Cohort (NHIS-HEALS) participated in this health screening program (Seong et al., 2017). The NHIS-HEALS includes a health screening program called the National Screening Program for Transitional Ages (NSPTA), which was initiated in 2007 for those aged 40 and 66 years because they are regarded as middle-aged and older adults, respectively (Kim et al., 2012). The NSPTA includes comprehensive questionnaires regarding medical history and cognitive status.

Additionally, the NSPTA, which is conducted with a 66-year-old population, contains a questionnaire on SCD as assessed by the Prescreening Korean Dementia Screening Questionnaire (KDSQ-P) (Jeon et al., 2010). The KDSQ-P is a 5-item self-reported questionnaire using a 3-Likert type scale (0 = no, 1 = yes, sometimes = 2 = yes, often). The five questions were as follows: Item 1, “Do you think that your memory is worse than that of your peers/friends?” Item 2, “Do you think your memory is worse than last year?”; Item 3, “Does your memory decline impact important activities or work?” Item 4, “Do others notice your memory decline?,” and item 5, “Do you think that you can no longer function as well as before due to your memory decline?” The total KDSQ-P score was distributed between 0 and 10, and participants with an overall score of 4 or higher were considered to have significant SCD. The aforementioned data sources have been described in detail in previous studies (Jeon et al., 2010; Kim et al., 2017; Seong et al., 2017).

### 2.2 Study cohort

#### 2.2.1 Definition of cognitive status

**FIGURE 1:**
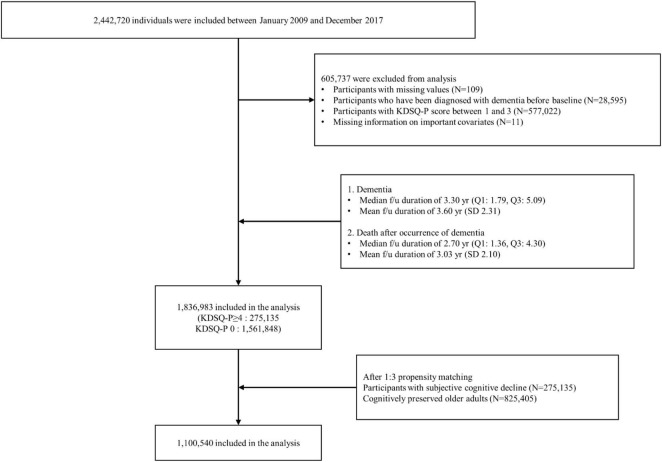
Flow chart depicting creation of study cohorts.

All participants aged 66 years who participated in the NSPTA between 2009 and 2017 were included in the study. Of the 2,442,720 NSPTA participants, we excluded those with missing values (*n* = 109), those who had been diagnosed with dementia before baseline (*n* = 28,595), those with KDSQ-P scores between 1 and 3 (*n* = 577,022), and those with missing information on important covariates (*n* = 11) (Figure 1). A total of 1,836,983 participants were included in the final analysis. CP (*n* = 1,561,848) was defined as subjects with a KDSQ-P score of 0, no history of ICD-10 (10th revision of the International Statistical Classification of Diseases and Related Health Problems) dementia codes (F00, F01, F02, F03, G30, F051, or G311), and no history of prescription of acetylcholinesterase inhibitors (donepezil, rivastigmine, and galantamine) or NMDA (N-methyl-D-aspartate) receptor antagonists at the time of NSPTA participation. SCD subjects (*n* = 275,135) were defined as subjects with a KDSQ-P score of 4 or more, because the cut-off point has been reported to be appropriate for detecting a person who needs a screening test for dementia in the validation study of the KDSQ-P (Jeon et al., 2010); no history of ICD-10 dementia code (F00, F01, F02, F03, G30, F051, or G311); and no history of prescription of acetylcholinesterase inhibitors (donepezil, rivastigmine, and galantamine) or NMDA receptor antagonists at the time of NSPTA participation. Supplementary methods provide a definition of demographic characteristics and medical history, including diabetes mellitus (DM), hypertension, ischemic heart disease, dyslipidemia, and depression. The definition of medical history has been described in detail in a previous study (Kim et al., 2018). The distribution of the sum of KDSQ-P scores in each group is summarized in Supplementary Table 1. This study was approved by the Institutional Review Board of Seoul St. Mary’s Hospital, Seoul, Korea (KC21ZISI0012). Consent from individual participants was not required because the study used publicly available anonymous data.

#### 2.2.2 Propensity matching

To address the issue of non-randomized treatment allocation, we employed a propensity score to identify matched patients from a pool of 1,561,848 cognitively preserved participants. Utilizing propensity score adjustments provides researchers with the capacity to ensure group comparability by evenly distributing biases and potential confounders (Baek et al., 2015). This method is highly regarded for its ability to equilibrate measured variables across groups prior to the analysis (Gayat et al., 2012).

Initially, we gathered the fundamental attributes of the 275,135 participants from the SCD group. Following this, we calculated the propensity of each SCD participant using a comprehensive unconditional logistic regression approach. Subsequently, the nearest neighbor matching technique was applied, and every SCD participant was paired with an individual from the CP group based on the closest propensity score (Austin, 2008). Both age and sex were incorporated as influential variables with a matching ratio of 1:3. This resulted in a total of 825,405 participants in the CP group. Given the vast size of our cohort, we established the caliper width (the acceptable difference in propensity scores for matched pairs) at 0 on the propensity score logit (Austin, 2011).

#### 2.2.3 Exposure variable—Infection

We evaluated the infection history before the NSPTA and investigated four infectious agents that have been reported to increase the risk of incident dementia since 2007: (1) *H. pylori*, (2) HSV, (3) VZV, and (4) HPV. We selected these four infectious agents for investigation, not only because of the frequent reports associating them with an increased risk of dementia onset but also because their potential mechanisms contributing to dementia have been elucidated in translational research. We classified participants into the infected group if they had a history of infection with any of the four infectious agents before the age of 66 since 2007. In addition, we categorized subjects into the non-infected group if they had no history of infection with any of the four infectious agents before the age of 66 years since 2007. Given these four types of infection sources, a single quadruple infection is possible. The number of subjects with quadruple infections in the CP and SCD groups was minor (NC group, *n* = 282; SCD group, *n* = 132). The infection monitoring period ranged from 2 to 10 years. Given the delay in diagnosing AD and the insidious nature of outcome measures, a 2-year “lag period” has been proposed, and studies have been conducted to assess the impact of infectious burden on the risk of AD (Rothman, 1981; Livingston et al., 2017; Douros et al., 2021). Therefore, for the purposes of this study, a duration of infection of 2 years or more can be considered a significant burden of infectious disease. Each infection was defined as follows: (1) *H. pylori* infection was defined as a history of prescription of *H. pylori* eradication medications (details for all eligible *H. pylori* eradication regimens are shown in Supplementary Table 2) with ICD-10 peptic ulcer codes (K25 and K26). These drug combinations were prescribed within the same prescription order, and the duration of therapy was between 7 and 14 days; (2) HSV infection was defined as an ICD-10 HSV code (B00) with at least two outpatient visits or one inpatient visit for symptomatic HSV infections according to the ICD-10 codes; (3) VZV infection was defined as an ICD-10 VZV code (B02) with at least two outpatient visits or one inpatient visit for symptomatic HSV infections according to these ICD-10 codes; and (4) HPV infection was defined as ICD-10 codes of anogenital (A630) or viral warts (B07) and HPV as the cause of diseases classified elsewhere (B97.7) with at least two outpatient visits or one inpatient visit for symptomatic HPV infections according to the ICD-10 codes. Additionally, when HSV and VZV are included in multiple infectious agents, HSV infection followed by VZV infection and VZV infection followed by HSV infection were categorized separately based on previous animal studies showing that quiescent HSV can be activated by VZV, contributing to a neuronal inflammatory reaction (Cairns et al., 2022).

#### 2.2.4 Outcome variable—Incident dementia

The incidence of dementia was defined by a concurrent diagnosis of dementia (based on ICD-10 codes F00, G30, F01, F02, F03, G23.1, G31.0, G31.1, G31.82, G31.83, G31.88, and F10.7) and the prescription of anti-dementia medication. The anti-dementia medications include an acetylcholinesterase inhibitor (rivastigmine, galantamine, or donepezil) or an N-methyl-D-aspartate receptor antagonist (memantine), which are most commonly used to treat dementia (O’Brien et al., 2017). Patients with dementia were grouped into AD (ICD-10 codes F00 and G30) or vascular dementia (VaD) (ICD-10 code F01) based on the diagnosis code at the first visit. If the diagnoses of both AD and VaD were recorded at the first visit, we used the diagnosis at the second visit. In the KNHIS, the following criteria must be met for a patient with dementia to receive reimbursement for the prescription of either acetylcholinesterase inhibitors or NMDA receptor antagonists: (1) Mini-Mental State Examination (MMSE) score ≤ 26, and (2) Clinical Dementia Rating (CDR) ≥ 1 or Global Deterioration Scale ≥ 3.

### 2.3 Statistical analysis

All continuous variables were expressed as mean ± SD, and categorical data were presented as numbers (percentages). Study participant characteristics according to cognitive status were compared using the Student’s *t*-test for continuous variables and the *x*^2^ test for categorical variables. The years of follow-up were calculated from the time of NSPTA participation to the occurrence of incident dementia or 31 December 2020, whichever came first. Multivariate Cox proportional hazards regression analysis was conducted to evaluate hazard ratios (HRs) according to cognitive status (SCD vs. CP) in each subcategory of infection (non-infection, single infection, dual infection, and triple infection), with the CP group as a reference category. Additionally, multivariate Cox proportional hazards regression analysis was performed to identify HRs for overall dementia, dementia due to AD, and VaD according to the infection categorized by the number of infectious agents (overall infection, single infection, dual infection, and triple infection), with non-infection as a reference category in each CP, SCD, and total group. Moreover, to clarify the effect of the number of infectious agents, a multivariate Cox proportional hazards regression analysis was performed to identify the HRs of overall dementia, dementia due to AD, and VaD according to the number of infectious agents, with a single infection as a reference category in each CP and SCD group. For calculating the “*p* for trend,” we treated the number of infections (categorized as single, dual, or triple infections) as an ordinal variable in our regression model. We then conducted a hypothesis test to determine whether there was a linear trend in the risk of developing dementia across infection categories for each cognitive status. The *p*-value for trend was calculated using a Cochran-Armitage trend test, which tested the null hypothesis that the differences in proportions of the risk of developing dementia across the number of infections were not significant. Statistical significance was considered at *p* < 0.05, indicating a significant trend in the risk of developing dementia across infection categories. Model 1 was not adjusted, Model 2 was adjusted for age and sex, and Model 3 was additionally adjusted for DM, hypertension, ischemic heart disease, dyslipidemia, and depression (Patterson et al., 2007; Sahathevan, 2015). The proportional hazards assumption was tested for all the main effects in all groups. There was no evidence that the proportional hazard assumption was violated in the CP, SCD, or the total groups. Additionally, we performed further Cox regression analysis using a stepwise selection method to evaluate the impact of various covariates on the risk of overall dementia. The analysis consisted of two main steps:

1. Univariate Analysis: Each variable was individually included in the model to assess its independent effect on dementia risk. This step helped determine the significance of each variable on its own.
2. Stepwise Selection: After fitting the full model, we applied the stepwise selection method to identify significant variables. This method combines forward selection and backward elimination, with a significance level of 0.05 for entry and stay in the model.

Moreover, To evaluate the model fit, we used the following statistics:

1. Akaike Information Criterion (AIC): AIC is used to compare models by balancing model fit and complexity. Lower AIC values indicate a better-fitting model.
2. Schwarz Bayesian Criterion (SBC) or Bayesian Information Criterion (BIC): Similar to AIC, BIC also considers model complexity but imposes a larger penalty for models with more parameters. Lower BIC values indicate a better-fitting model.

These statistics help determine the overall quality of the model and guide the selection of significant covariates by assessing how well the model explains the data while accounting for the number of predictors included. For all statistical analyses, we used SAS version 9.3 (SAS Institute, Cary, NC, USA), with *p*-values < 0.05 considered significant.

## 3 Results

### 3.1 Participant characteristics

After PSM, 1,100,540 patients were included in the final analysis. Among them, 825,405 and 275,135 participants were categorized into the CP and SCD groups, respectively (Figure 1). Table 1 summarizes the baseline characteristics of study participants after PSM. There was a significant difference in infection history between the CP and SCD groups. The SCD group exhibited a higher infection rate than the CP group regardless of the number of infectious agents. Furthermore, the infection rate decreased in both groups as the number of infectious agents increased. Additionally, the proportion of patients with DM, hypertension, ischemic heart disease, dyslipidemia, and depression was consistently higher in the SCD group than in the CP group.

**TABLE 1:** Baseline characteristics of the study participants.

	CP (*n* = 825,405)	SCD (*n* = 275,135)	*p*
Age (mean ± SD)	67.34 ± 2.22	67.34 ± 2.22	1.0000
Sex [*n* (% of male)]	341,229 (41.3%)	113,743 (41.3%)	1.0000
Sum of KDSQ-P score (mean ± SD)	0 ± 0	5.04 ± 1.33	< 0.0001
**Infection history [*n* (%)]**			
Non-infection [*n* (%)]	512,026 (62.0%)	169,241 (61.5%)	< 0.0001
Single infection [*n* (%)]	233,732 (28.3%)	78,116 (28.4%)	
*H. pylori*	53,273 (6.5%)	17,890 (6.5%)	
HSV	75,144 (9.1%)	25,093 (9.1%)	
VZV	94,558 (11.5%)	31,444 (11.4%)	
HPV	10,757 (1.3%)	3,689 (1.3%)	
Dual infection [*n* (%)]	71,213 (8.6%)	24,546 (8.9%)	
*H. pylori* + HSV	9,225 (1.1%)	3,163 (1.1%)	
*H. pylori* + VZV	11,051 (1.3%)	3,843 (1.4%)	
*H. pylori* + HPV	1,406 (0.17%)	473 (0.17%)	
HSV + VZV*	17,131 (2.08%)	5,882 (2.14%)	
VZV + HSV^†^	27,107 (3.28%)	9,289 (3.38%)	
VZV + HSV^‡^	44,238 (5.36%)	15,171 (5.51%)	
HSV + HPV	2,620 (0.32%)	941 (0.34%)	
VZV + HPV	2,673 (0.32%)	955 (0.35%)	
Triple infection [*n* (%)]	8,152 (0.99%)	3,100 (1.13%)	
*H. pylori* + HSV + VZV*	2,191 (0.27%)	864 (0.31%)	
*H. pylori* + VZV + HSV^†^	3,428 (0.42%)	1,217 (0.44%)	
*H. pylori* + VZV + HSV^‡^	5,619 (0.68%)	2,081 (0.76%)	
*H. pylori* + HSV + HPV	378 (0.05%)	152 (0.06%)	
*H. pylori* + VZV + HPV	374 (0.05%)	152 (0.06%)	
HSV + VZV + HPV*	748 (0.09%)	293 (0.11%)	
VZV + HSV + HPV^†^	1,033 (0.13%)	422 (0.15%)	
VZV + HSV + HPV^‡^	1,781 (0.22%)	715 (0.26%)	
Quadruple infection [*n* (%)]	282 (0.03%)	132 (0.05%)	
H. pyroli + HSV + VZV + HPV*	116 (0.01%)	52 (0.02%)	
H. pyroli + VZV + HSV + HPV^†^	166 (0.02%)	80 (0.03%)	
H. pyroli + HSV + VZV + HPV^‡^	282 (0.03%)	132 (0.05%)	
Follow-up years (mean ± SD)	5.37 ± 1.96	5.49 ± 2.23	< 0.0001
Diabetes [*n* (%)]	316,822 (38.4%)	115,152 (41.9%)	< 0.0001
Hypertension [*n* (%)]	503,246 (61.0%)	169,493 (61.6%)	< 0.0001
Ischemic heart disease [*n* (%)]	172,700 (20.9%)	68,874 (25.0%)	< 0.0001
Dyslipidemia [*n* (%)]	544,725 (66.0%)	190,273 (69.2%)	< 0.0001
Depression [*n* (%)]	154,282 (18.7%)	72,840 (26.5%)	< 0.0001

*P*-value by *t*-test for continuous variables and by *x*^2^ test for categorical variables. CP, cognitively preserved older adults; SCD, subjective cognitive decline; KDSQ-P, Prescreening Korean Dementia Screening Questionnaire; *H. pylori*, *Helicobacter pylori*; HSV, herpes simplex virus; VZV, varicella zoster virus; HPV, human papillomavirus; SD, standard deviation;

*, HSV infection followed by VZV infection;

^†^VZV infection followed by HSV infection;

^‡^infection order not considered.

### 3.2 Risk of incident dementia according to cognitive status in subgroups categorized by number of infectious agents

**FIGURE 2:**
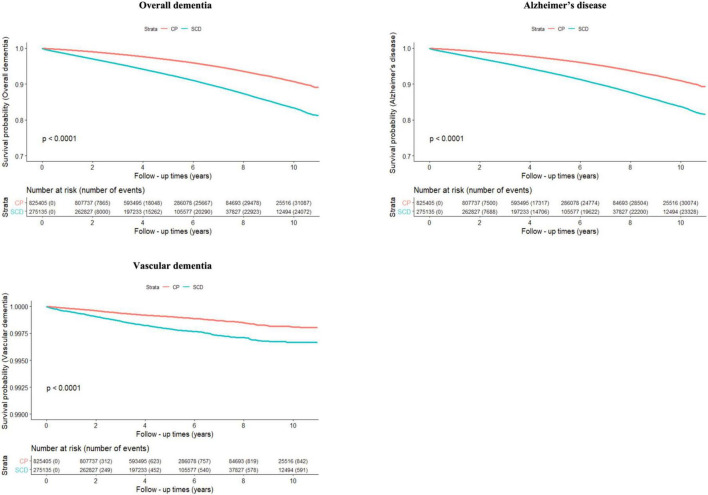
Cumulative risk of incident dementia for cognitively preserved group (CP) and participants with subjective cognitive decline (SCD). The results shown are survival probability curves for overall dementia, dementia due to Alzheimer’s disease, and vascular dementia as a function of follow-up times in the cognitively preserved and subjective cognitive decline groups, with covariates unadjusted (Model 1). CP, cognitively preserved; SCD, subjective cognitive decline.

Figure 2 and Table 2 show the risk of incident dementia according to cognitive status. The risk of overall dementia significantly increased by approximately two-fold in the SCD group compared with the CP group, irrespective of the number of infectious agents, after controlling for the covariates of Model 3 (non-infection, aHR = 2.075, 95% CI = 2.018–2.135; single infection, aHR = 2.035, 95% CI = 1.948–2.127; dual infection, aHR = 2.049, 95% CI = 1.889–2.223; triple infection, aHR = 1.958, 95% CI = 1.553–2.468; quadruple infection, aHR = 6.888, 95% CI = 1.194–39.754). The risk of dementia due to AD was approximately twice as high in the SCD group as in the CP group, regardless of the number of infectious agents, after adjusting for the covariates of Model 3 (non-infection, aHR = 2.081, 95% CI = 2.022–2.141; single infection, aHR = 2.049, 95% CI = 1.96–2.143; dual infection, aHR = 2.028, 95% CI = 1.867–2.203; triple infection, aHR = 1.98, 95% CI = 1.568–2.5; quadruple infection, aHR = 6.888, 95% CI = 1.194–39.754). The risk for VaD also increased in the SCD group compared to that in the CP group in the non- to dual-infection groups after adjusting for the covariates of Model 3 (non-infection, aHR = 1.9, 95% CI = 1.604–2.251; single infection, aHR = 1.569, 95% CI = 1.19–2.069; dual infection, aHR = 2.782, 95% CI = 1.644–4.706), and no significant risk change was observed in the triple-infection group.

**TABLE 2:** Risk of incident dementia according to cognitive status in subgroups categorized by number of infectious agents.

Infection history	Cognitive status	Number	Overall dementia	Duration (person years)	IR per 1000	Unadjusted HR (95% CI) (Model 1)			Adjusted HR (95% CI) (Model 2)			Adjusted HR (95% CI) (Model 3)		
						**Hazard ratio**	**Low**	**High**	**Hazard ratio**	**Low**	**High**	**Hazard ratio**	**Low**	**High**
Non-infection	CP	512,026	20,261	2,851,925.38	7.1	1 (ref.)			1 (ref.)			1 (ref.)		
	SCD	169,241	15,690	967,844.90	16.2	2.231	2.185	2.278	2.247	2.201	2.295	2.075	2.018	2.135
Single infection	CP	233,732	8,310	1,197,071.41	6.9	1 (ref.)			1 (ref.)			1 (ref.)		
	SCD	78,116	6,402	407,900.79	15.7	2.221	2.149	2.294	2.217	2.146	2.291	2.035	1.948	2.127
Dual infection	CP	71,213	2,447	343,275.51	7.1	1 (ref.)			1 (ref.)			1 (ref.)		
	SCD	24,546	1,904	119,804.97	15.9	2.201	2.073	2.337	2.181	2.054	2.315	2.049	1.889	2.223
Triple infection	CP	8,152	294	37,479.16	7.8	1 (ref.)	1 (ref.)	1 (ref.)						
	SCD	3,100	245	14,163.96	17.3	2.187	1.846	2.591	2.154	1.817	2.552	1.958	1.553	2.468
Quadruple infection	CP	282	6	1,241.51	4.8	1 (ref.)			1 (ref.)			1 (ref.)		
	SCD	132	10	568.13	17.6	3.714	1.349	10.23	4.372	1.577	12.12	6.888	1.194	39.754
**Infection history**	**Cognitive status**	**Number**	**Alzheimer’s disease**	**Duration (person years)**	**IR per 1000**	**Unadjusted HR (95% CI) (Model 1)**			**Adjusted HR (95% CI) (Model 2)**			**Adjusted HR (95% CI) (Model 3)**		
						**Hazard ratio**	**Low**	**High**	**Hazard ratio**	**Low**	**High**	**Hazard ratio**	**Low**	**High**
Non-infection	CP	512,026	19,575	2,851,925.38	6.9	1 (ref.)			1 (ref.)			1 (ref.)		
	SCD	169,241	15,181	967,844.90	15.7	2.232	2.186	2.28	2.249	2.202	2.298	2.081	2.022	2.141
Single infection	CP	233,732	8,037	1,197,071.41	6.7	1 (ref.)			1 (ref.)			1 (ref.)		
	SCD	78,116	6,229	407,900.79	15.3	2.232	2.16	2.308	2.229	2.156	2.304	2.049	1.96	2.143
Dual infection	CP	71,213	2,396	343,275.51	7.0	1 (ref.)			1 (ref.)			1 (ref.)		
	SCD	24,546	1,846	119,804.97	15.4	2.179	2.051	2.316	2.159	2.031	2.294	2.028	1.867	2.203
Triple infection	CP	8,152	287	37,479.16	7.7	1 (ref.)	1 (ref.)	1 (ref.)						
	SCD	3,100	240	14,163.96	16.9	2.193	1.848	2.604	2.159	1.819	2.563	1.98	1.568	2.5
Quadruple infection	CP	282	6	1,241.51	4.8	1 (ref.)			1 (ref.)			1 (ref.)		
	SCD	132	10	568.13	17.6	3.714	1.349	10.23	4.372	1.577	12.12	6.888	1.194	39.754
**Infection history**	**Cognitive status**	**Number**	**Vascular dementia**	**Duration (person years)**	**IR per 1000**	**Unadjusted HR (95% CI) (Model 1)**			**Adjusted HR (95% CI) (Model 2)**			**Adjusted HR (95% CI) (Model 3)**		
						**Hazard ratio**	**Low**	**High**	**Hazard ratio**	**Low**	**High**	**Hazard ratio**	**Low**	**High**
Non-infection	CP	512,026	555	2,851,925.38	0.2	1 (ref.)			1 (ref.)			1 (ref.)		
	SCD	169,241	394	967,844.90	0.4	2.109	1.853	2.4	2.113	1.857	2.404	1.9	1.604	2.251
Single infection	CP	233,732	237	1,197,071.41	0.2	1 (ref.)			1 (ref.)			1 (ref.)		
	SCD	78,116	143	407,900.79	0.4	1.787	1.452	2.2	1.786	1.451	2.198	1.569	1.19	2.069
Dual infection	CP	71,213	45	343,275.51	0.1	1 (ref.)			1 (ref.)			1 (ref.)		
	SCD	24,546	49	119,804.97	0.4	3.112	2.075	4.665	3.097	2.066	4.644	2.782	1.644	4.706
Triple infection	CP	8,152	6	37,479.16	0.2	1 (ref.)	1 (ref.)	1 (ref.)						
	SCD	3,100	5	14,163.96	0.4	2.237	0.683	7.331	2.248	0.686	7.639	0.841	0.085	8.338
Quadruple infection	CP	282	0	1,241.51	0.0	1 (ref.)			1 (ref.)			1 (ref.)		
	SCD	132	0	568.13	0.0	0	0	NA	0	0	NA	0	0	NA

The results shown are hazard ratios and 95% confidence intervals, with unadjusted HRs (Model 1), HRs adjusted for age and sex (Model 2), and additionally adjusted for diabetes mellitus, hypertension, ischemic heart disease, dyslipidemia, and depression (Model 3). CP, cognitively preserved older adults; SCD, subjective cognitive decline; IR, incidence rate; HR, hazard ratio; CI, confidence interval; NA, non-applicable.

### 3.3 Risk of incident dementia according to infection in each group categorized by cognitive status

The incidence rates (IRs) and HRs for incident dementia according to infection are shown in Table 3. When considering the participants as a whole, the infection group had a higher risk of overall dementia and dementia due to AD than the non-infection group (overall dementia, unadjusted HR = 1.034, 95% CI = 1.016–1.052; AD, unadjusted HR = 1.04, 95% CI = 1.022–1.059). However, after adjusting for covariates in Model 3, the infection group’s risk of both overall dementia and dementia due to AD showed a decrease compared to the non-infection group (overall dementia, adjusted HR = 0.899, 95% CI = 0.878–0.92; AD, adjusted HR = 0.903, 95% CI = 0.882–0.925). In the CP group, the risk of overall dementia and dementia due to AD was higher in the infection group than in the non-infection group (overall dementia: unadjusted HR = 1.051, 95% CI = 1.027–1.076; AD: unadjusted HR = 1.057, 95% CI = 1.032–1.083). However, after adjusting for covariates in Model 3, we observed no significant change in the risk of overall dementia between the two groups, and the risk of dementia due to AD was lower in the infection group than that in the non-infection group (AD, adjusted HR = 0.914, 95% CI = 0.885–0.944). In the SCD group, no significant risk difference was observed in the unadjusted model. After covariate adjustment in Model 3, the risks of overall dementia, dementia due to AD, and VaD were lower in the infection group than in the non-infection group (overall dementia: adjusted HR = 0.88, 95% CI = 0.849–0.912; AD: aHR = 0.885, 95% CI = 0.854–0.918; VaD: aHR = 0.786, 95% CI = 0.625–0.989).

**TABLE 3:** Risk of incident dementia according to infection in **(A)** total participants, **(B)** cognitively preserved older adults, and **(C)** participants with subjective cognitive decline.

(A) Total participants.													
**Infection history**	**Number**	**Overall dementia**	**Duration (person years)**	**IR per 1000**	**Unadjusted HR (95% CI) (Model 1)**			**Adjusted HR (95% CI) (Model 2)**			**Adjusted HR (95% CI) (Model 3)**		
					**Hazard ratio**	**Low**	**High**	**Hazard ratio**	**Low**	**High**	**Hazard ratio**	**Low**	**High**
Non-infection	681,257	35,951	3,819,770.28	9.4	1 (ref.)			1 (ref.)			1 (ref.)		
Infection	419,273	19,618	2,121,505.45	9.2	1.034	1.016	1.052	0.988	0.971	1.005	0.899	0.878	0.92
**Infection history**	**Number**	**Alzheimer’s disease**	**Duration (person years)**	**IR per 1000**	**Unadjusted HR (95% CI)k (Model 1)**			**Adjusted HR (95% CI) (Model 2)**			**Adjusted HR (95% CI) (Model 3)**		
					**Hazard ratio**	**Low**	**High**	**Hazard ratio**	**Low**	**High**	**Hazard ratio**	**Low**	**High**
Non-infection	681,257	34,756	3,819,770.28	9.1	1 (ref.)			1 (ref.)			1 (ref.)		
Infection	419,273	19,051	2,121,505.45	9.0	1.04	1.022	1.059	0.993	0.975	1.011	0.903	0.882	0.925
**Infection history**	**Number**	**Vascular dementia**	**Duration (person years)**	**IR per 1000**	**Unadjusted HR (95% CI) (Model 1)**			**Adjusted HR (95% CI) (Model 2)**			**Adjusted HR (95% CI) (Model 3)**		
					**Hazard ratio**	**Low**	**High**	**Hazard ratio**	**Low**	**High**	**Hazard ratio**	**Low**	**High**
Non-infection	681,257	949	3,819,770.28	0.2	1 (ref.)			1 (ref.)			1 (ref.)		
Infection	419,273	485	2,121,505.45	0.2	0.905	0.811	1.01	0.91	0.815	1.016	0.828	0.716	0.957
**(B) Cognitively preserved older adults.**													
**Infection history**	**Number**	**Overall dementia**	**Duration (person years)**	**IR per 1000**	**Unadjusted HR (95% CI) (Model 1)**			**Adjusted HR (95% CI) (Model 2)**			**Adjusted HR (95% CI) (Model 3)**		
					**Hazard ratio**	**Low**	**High**	**Hazard ratio**	**Low**	**High**	**Hazard ratio**	**Low**	**High**
Non-infection	512,026	20,261	2,851,925.38	7.1	1 (ref.)			1 (ref.)			1 (ref.)		
Infection	313,379	11,057	1,579,067.60	7.0	1.051	1.027	1.076	0.996	0.973	1.019	0.856	0.71	1.033
**Infection history**	**Number**	**Alzheimer’s disease**	**Duration (person years)**	**IR per 1000**	**Unadjusted HR (95% CI)k (Model 1)**			**Adjusted HR (95% CI) (Model 2)**			**Adjusted HR (95% CI) (Model 3)**		
					**Hazard ratio**	**Low**	**High**	**Hazard ratio**	**Low**	**High**	**Hazard ratio**	**Low**	**High**
Non-infection	512,026	19,575	2,851,925.38	6.9	1 (ref.)			1 (ref.)			1 (ref.)		
Infection	313,379	10,726	1,579,067.60	6.8	1.057	1.032	1.083	1	0.977	1.024	0.914	0.885	0.944
**Infection history**	**Number**	**Vascular dementia**	**Duration (person years)**	**IR per 1000**	**Unadjusted HR (95% CI) (Model 1)**			**Adjusted HR (95% CI) (Model 2)**			**Adjusted HR (95% CI) (Model 3)**		
					**Hazard ratio**	**Low**	**High**	**Hazard ratio**	**Low**	**High**	**Hazard ratio**	**Low**	**High**
Non-infection	512,026	555	2,851,925.38	0.2	1 (ref.)			1 (ref.)			1 (ref.)		
Infection	313,379	288	1,579,067.60	0.2	0.932	0.808	1.076	0.932	0.807	1.075	0.856	0.71	1.033
**(C) Participants with subjective cognitive decline.**													
**Infection history**	**Number**	**Overall dementia**	**Duration (person years)**	**IR per 1000**	**Unadjusted HR (95% CI) (Model 1)**			**Adjusted HR (95% CI) (Model 2)**			**Adjusted HR (95% CI) (Model 3)**		
					**Hazard ratio**	**Low**	**High**	**Hazard ratio**	**Low**	**High**	**Hazard ratio**	**Low**	**High**
Non-infection	169,241	15,690	967,844.90	16.2	1 (ref.)			1 (ref.)			1 (ref.)		
Infection	105,894	8,561	542,437.85	15.8	1.001	0.975	1.028	0.964	0.939	0.99	0.88	0.849	0.912
**Infection history**	**Number**	**Alzheimer’s disease**	**Duration (person years)**	**IR per 1000**	**Unadjusted HR (95% CI)k (Model 1)**			**Adjusted HR (95% CI) (Model 2)**			**Adjusted HR (95% CI) (Model 3)**		
					**Hazard ratio**	**Low**	**High**	**Hazard ratio**	**Low**	**High**	**Hazard ratio**	**Low**	**High**
Non-infection	169,241	15,181	967,844.90	15.7	1 (ref.)			1 (ref.)			1 (ref.)		
Infection	105,894	8,325	542,437.85	15.3	1.008	0.981	1.035	0.969	0.943	0.996	0.885	0.854	0.918
**Infection history**	**Number**	**Vascular dementia**	**Duration (person years)**	**IR per 1000**	**Unadjusted HR (95% CI) (Model 1)**			**Adjusted HR (95% CI) (Model 2)**			**Adjusted HR (95% CI) (Model 3)**		
					**Hazard ratio**	**Low**	**High**	**Hazard ratio**	**Low**	**High**	**Hazard ratio**	**Low**	**High**
Non-infection	169,241	394	967,844.90	0.4	1 (ref.)			1 (ref.)			1 (ref.)		
Infection	105,894	197	542,437.85	0.4	0.86	0.725	1.021	0.87	0.732	1.033	0.786	0.625	0.989

The results shown are hazard ratios and 95% confidence intervals, with unadjusted HRs (Model 1), HRs adjusted for age and sex (Model 2), and additionally adjusted for diabetes mellitus, hypertension, ischemic heart disease, dyslipidemia, and depression (Model 3). IR, incidence rate; HR, hazard ratio; CI, confidence interval.

### 3.4 Risk of incident dementia according to the infection stratified by the number of infectious agents across cognitive status

**FIGURE 3:**
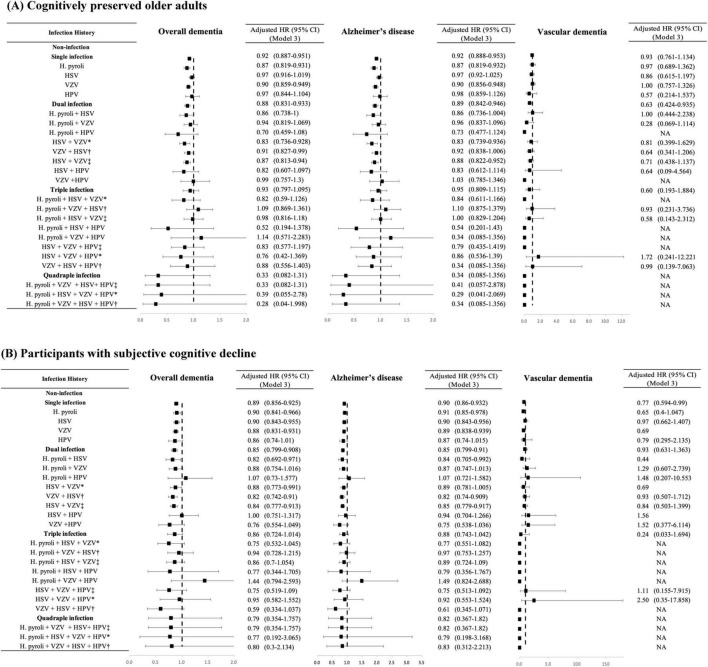
Risk of incident dementia according to the number and the type of infectious agents in each group categorized by cognitive status. **(A)** Cognitively preserved older adults. **(B)** Participants with subjective cognitive decline. The results are shown as hazard ratios and 95% confidence intervals, with HRs adjusted for age, sex, diabetes mellitus, hypertension, ischemic heart disease, dyslipidemia, and depression (Model 3). HR, hazard ratio; CI, confidence interval; *H. pylori*, *Helicobacter pylori*; HSV, herpes simplex virus; VZV, varicella zoster virus; HPV, human papillomavirus; SD, standard deviation; * HSV infection followed by VZV infection; ^†^VZV infection followed by HSV infection; ^‡^infection order not considered. NA, non-applicable.

The aHRs for the association between infection categories (single, dual, and triple infections) and the risk of incident dementia stratified by cognitive status groups are shown in Figure 3. In the CP group, the risks for overall dementia and dementia due to AD were significantly higher in subjects with single to triple infections than in those without infections in the unadjusted analyses (Supplementary Table 3). However, after controlling for the covariates of Model 3, subjects with single and dual infections showed lower risks for both overall dementia (single infection, adjusted HR = 0.919, 95% CI = 0.887–0.951; dual infection, adjusted HR = 0.881, 95% CI = 0.831–0.933) and dementia due to AD (single infection, adjusted HR = 0.92, 95% CI = 0.888–0.953; dual infection, adjusted HR = 0.892, 95% CI = 0.842–0.946) compared to the non-infection group, and no significant risk change was observed in those with triple infection. Additionally, while dual infection with HSV and VZV was associated with a higher risk of overall dementia and dementia due to AD than in non-infected individuals when the order of infection was not considered, the unadjusted HR was lower when HSV infection was followed by VZV infection than in the reverse sequence. Furthermore, in the triple infection group, which included *H. pylori* infection in addition to HSV and VZV, there was an increased risk of overall dementia and dementia due to AD compared to the non-infection group when VZV infection occurred before HSV infection, whereas no significant change in risk was observed when HSV infection occurred before VZV infection in an unadjusted model. After adjusting for the covariates of Model 3, in cases of dual infection with HSV and VZV, the risk of overall dementia and AD decreased compared with that in the non-infection group, regardless of the order of infection. In the case of triple infections with *H. pylori*, HSV, and VZV, after adjusting for covariates in Model 3, no significant changes were observed in the risk of overall dementia and AD irrespective of the infection order. In cases of quadruple infection, no significant change in risk was observed for both overall dementia and AD, and no events of vascular dementia were reported.

In the SCD group, the risk of dementia due to AD was higher in subjects with triple infection than in those without infection (unadjusted HR = 1.14, 95% CI 1.003–1.295). However, after controlling for covariates in Model 3, there was no significant change in risk in the triple infection group compared to that in the non-infection group. The order of HSV and VZV infection did not have a significant impact on the results in the SCD group.

Table 4 presents the IRs and HRs for incident dementia according to the number of infectious agents, with a single infection as a reference category. In the CP group, we observed a notable increase in the IR per 1,000 individuals with overall dementia and dementia due to AD as the number of infections increased from single to triple, indicating a significant upward trend (*p* for trend < 0.001). In the SCD group, similar to the results of the CP group, a remarkable increase in the IR per 1,000 individuals with overall dementia and dementia due to AD was detected as the number of infectious agents increased, which demonstrated a significant upward trend (*p* for trend < 0.001). These findings show a positive dose-response relationship between the frequency of infections and the risk of developing dementia in both the CP and SCD groups. In addition, in the CP group, an increasing number of infections was associated with higher unadjusted HRs for overall dementia (dual infection, unadjusted HR = 1.064, 95% CI = 1.017–1.113; triple infection, unadjusted HR = 1.204, 95% CI = 1.072–1.352) and dementia due to AD (dual infection, unadjusted HR = 1.079, 95% CI = 1.031–1.129; triple infection, unadjusted HR = 1.218, 95% CI = 1.082–1.37) compared than in the single infection group. However, after adjusting for covariates in Model 3, no significant change in risk was observed for individuals with dual and triple infections compared with those with a single infection. In the SCD group, individuals with triple infections demonstrated an increased risk of dementia due to AD compared with those with a single infection (unadjusted HR = 1.139, 95% CI 1.001–1.296). However, after controlling for covariates in Model 3, there was no significant change in risk in the triple infection group compared with that in the single infection group. In both the unadjusted and adjusted models, there was no significant increase in the risk of VaD in the dual- and triple-infection groups compared to the single-infection group, regardless of cognitive status. In the CP group, dual infections had 45 cases of VaD and triple infections had 6 cases, both of which were fewer than the overall dementia and dementia due to AD incidence. Similarly, in the SCD group, there were 49 and 5 cases of VaD for dual and triple infections, respectively, which were lower than the numbers for overall dementia and dementia due to AD. In the case of quadruple infections, only AD events were observed, with 6 cases in the CP group and 10 cases in the SCD group. The IR per 1,000 was the highest in the SCD group, but in the CP group, quadruple infection deviated from the increasing trend of IR per 1,000 as the number of infectious agents increased. HRs did not show significant change in risk in both unadjusted and adjusted models.

**TABLE 4:** Risk of incident dementia according to number of infectious agents in subgroups categorized by cognitive status.

(A) Overall dementia.															
**Infection history**	**Cognitive status**	**Number**	**Overall dementia**	**Duration (person years)**	**IR per 1000**	***p* for trend**	**Unadjusted HR (95% CI) (Model 1)**			**Adjusted HR (95% CI) (Model 2)**			**Adjusted HR (95% CI) (Model 3)**		
							**Hazard ratio**	**Low**	**High**	**Hazard ratio**	**Low**	**High**	**Hazard ratio**	**Low**	**High**
Single infection	CP	233,732	8,310	1,197,071.41	6.9	< 0.0001	1 (ref.)			1 (ref.)			1 (ref.)		
Dual infection		71,213	2,447	343,275.51	7.1		1.064	1.017	1.113	1.022	0.976	1.069	0.953	0.896	1.014
Triple infection		8,152	294	37,479.16	7.8		1.204	1.072	1.352	1.145	1.019	1.286	1.013	0.863	1.189
Quadruple infection		282	6	1,241.51	4.8		0.756	0.34	1.683	0.688	0.309	1.531	0.353	0.088	1.41
Single infection	SCD	78,116	6,402	407,900.79	15.7	< 0.0001	1 (ref.)			1 (ref.)			1 (ref.)		
Dual infection		24,546	1,904	119,804.97	15.9		1.027	0.975	1.081	1	0.95	1.053	0.954	0.891	1.021
Triple infection		3,100	245	14,163.96	17.3		1.129	0.994	1.283	1.088	0.957	1.236	0.961	0.81	1.139
Quadruple infection		132	10	568.13	17.6		1.164	0.626	2.164	1.15	0.619	2.139	0.877	0.394	1.953
**(B) Alzheimer’s disease.**															
**Infection history**	**Cognitive status**	**Number**	**Alzheimer’s disease**	**Duration (person years)**	**IR per 1000**	***p* for trend**	**Unadjusted HR (95% CI) (Model 1)**			**Adjusted HR (95% CI) (Model 2)**			**Adjusted HR (95% CI) (Model 3)**		
							**Hazard ratio**	**Low**	**High**	**Hazard ratio**	**Low**	**High**	**Hazard ratio**	**Low**	**High**
Single infection	CP	233,732	8,037	1,197,071.41	6.71	< 0.0001	1 (ref.)			1 (ref.)			1 (ref.)		
Dual infection		71,213	2,396	343,275.51	6.98		1.079	1.031	1.129	1.035	0.988	1.083	0.964	0.906	1.027
Triple infection		8,152	287	37,479.16	7.66		1.218	1.082	1.37	1.157	1.028	1.302	1.028	0.874	1.209
Quadruple infection		282	6	1,241.51	4.83		0.784	0.352	1.745	0.712	0.32	1.585	0.365	0.091	1.458
Single infection	SCD	78,116	6,229	407,900.79	15.3	< 0.0001	1 (ref.)			1 (ref.)			1 (ref.)		
Dual infection		24,546	1,846	119,804.97	15.4		1.024	0.972	1.079	0.996	0.946	1.05	0.949	0.885	1.017
Triple infection		3,100	240	14,163.96	16.9		1.139	1.001	1.296	1.096	0.963	1.247	0.981	0.826	1.164
Quadruple infection		132	10	568.13	17.6		1.199	0.645	2.23	1.185	0.637	2.204	0.902	0.405	2.009
(C) Vascular dementia.															
**Infection history**	**Cognitive status**	**Number**	**Vascular dementia**	**Duration (person years)**	**IR per 1000**	***p* for trend**	**Unadjusted HR (95% CI) (Model 1)**			**Adjusted HR (95% CI) (Model 2)**			**Adjusted HR (95% CI) (Model 3)**		
							**Hazard ratio**	**Low**	**High**	**Hazard ratio**	**Low**	**High**	**Hazard ratio**	**Low**	**High**
Single infection	CP	233,732	237	1,197,071.41	0.20	0.0012	1 (ref.)			1 (ref.)			1 (ref.)		
Dual infection		71,213	45	343,275.51	0.13		0.66	0.479	0.907	0.655	0.476	0.901	0.677	0.447	1.025
Triple infection		8,152	6	37,479.16	0.16		0.803	0.357	1.806	0.792	0.352	1.781	0.645	0.205	2.028
Quadruple infection		282	0	1,241.51	0.00		0	0	2.21E+157	0	0	1.38E+157	0	0	3.19E+203
Single infection	SCD	78,116	143	407,900.79	0.35	0.0258	1 (ref.)			1 (ref.)			1 (ref.)		
Dual infection		24,546	49	119,804.97	0.41		1.14	0.824	1.578	1.151	0.831	1.593	1.183	0.776	1.803
Triple infection		3,100	5	14,163.96	0.35		0.967	0.396	2.36	0.972	0.398	2.373	0.303	0.042	2.179
Quadruple infection		132	0	568.13	0.00		0	0	7.50E+266	0	0	7.16E+266	0	0	NA

The results shown are hazard ratios and 95% confidence intervals, with unadjusted HRs (Model 1), HRs adjusted for age and sex (Model 2), and additionally adjusted for diabetes mellitus, hypertension, ischemic heart disease, dyslipidemia, and depression (Model 3). CP, cognitively preserved older adults; SCD, subjective cognitive decline; IR, incidence rate; HR, hazard ratio; CI, confidence interval; NA, non-applicable.

### 3.5 Stepwise selection results

#### 3.5.1 Univariate analysis

The results of the univariate analysis highlighted the significance of each variable, with all variables showing a *p*-value < 0.0001, indicating high significance. The HRs for each variable are presented in Supplementary Table 4A.

#### 3.5.2 Stepwise selection

The final model obtained through stepwise selection includes the following significant variables and their impact:

- Age: HR = 1.144 (95% CI: 1.139–1.150), *p* < 0.0001
- Sex (Female): HR = 1.147 (95% CI: 1.105–1.191), *p* < 0.0001
- DM: HR = 1.42 (95% CI: 1.385–1.455), *p* < 0.0001
- Dyslipidemia: HR = 0.963 (95% CI: 0.937–0.99), *p* = 0.0075
- Hypertension: HR = 1.19 (95% CI: 1.158–1.221), *p* < 0.0001
- Ischemic Heart Disease: HR = 1.136 (95% CI: 1.106–1.166), *p* < 0.0001
- Depression: HR = 2.302 (95% CI: 2.247–2.359), *p* < 0.0001

Depression emerged as the most influential variable, while dyslipidemia was found to be the least influential variable (Supplementary Tables 4B, C).

#### 3.5.3 Model fit statistics

- AIC: Lower values indicate a better model. The AIC decreased from 800,931.29 to 789,839.76, indicating an improved fit.
- BIC: The BIC decreased from 800,931.29 to 789,963.85, indicating a better-fitting model with the selected covariates.

These fit statistics indicate that the model with the selected covariates fits the data better than the model without covariates (Supplementary Table 4D).

## 4 Discussion

This study aimed to investigate the complex interplay between infections and dementia, with a specific focus on the influence of various types and numbers of infections on dementia onset in individuals with and without SCD. Utilizing a comprehensive, nationwide, population-based cohort, our research stands out because of its unprecedented scale and diversity, offering a thorough representation of the population. This robust approach enabled us to delve deeply into several key areas. First, we examined how SCD affects the onset of dementia in relation to the number of infectious agents. Second, we assessed the effects of infections on the development of dementia within both the CP and SCD groups. Third, we explored the comparative impact of single, dual, and triple infections on the risk of dementia across these cognitive statuses. The expansive scope of our cohort, covering the entire nation, provides a unique and comprehensive dataset, bolstering the reliability and applicability of our findings. Our analysis offers not only a clearer understanding of the infection-dementia nexus but also sheds light on epidemiological trends and risk factors for dementia. By leveraging this extensive data analysis, our study aimed to provide novel insights into the multifaceted nature of dementia, especially in understanding the interrelations between SCD, infections, and cognitive decline.

First, regarding the impact of SCD on the risk of dementia, our study provides compelling evidence. We meticulously analyzed the influence of cognitive status, particularly SCD, on the onset of dementia in the context of different infection statuses. Our findings demonstrate a significant trend: regardless of infection status and number of infectious agents, individuals with SCD showed a consistently elevated risk of developing both overall dementia and AD. With single to triple infections, this risk was approximately twofold higher than that in the CP group, and in cases of quadruple infection, it showed more than a sixfold increase in risk. Given the small sample size of quadruple infections, there is a possibility of inconsistent results. Although the risk of VaD also increased in individuals with SCD, this pattern did not show the same level of consistency as that observed in AD. This aligns with previous studies that have reported that older adults with SCD have an approximately doubled risk of dementia onset over an average follow-up period of 4.8 years (Mitchell et al., 2014). Another systematic review study also found that the SCD group had approximately twice the risk of progression to dementia compared to normal aging over a follow-up period of 5.27 years, which is consistent with the findings of our study (Parfenov et al., 2020). Our findings reinforce the notion that SCD is a critical factor for dementia risk, particularly emphasizing its strong association with AD.

Second, we assessed the effects of infection on the development of dementia in both the CP and SCD groups. Initially, in the unadjusted models, the infection group appeared to have an elevated risk of overall dementia and AD, especially in the CP cohort. However, after adjusting for established dementia risk covariates like DM (Ninomiya, 2014), hypertension (Perrotta et al., 2016), dyslipidemia (Reitz, 2013), and depression (Bennett and Thomas, 2014), this initially observed risk was found to be not significant or intriguingly indicated a potential protective effect. This contrast suggests a complex interplay among infections, individual health conditions, and dementia risk. A possible protective effect of infections against dementia observed in the adjusted results may be attributed to the influence of immunity on the onset of dementia. Existing studies suggest that an increase in adaptive immunity markers is associated with a reduced risk of developing dementia, which implies that infection-induced activation of adaptive immunity might confer a protective effect against dementia (Zhang et al., 2022). However, it is important not to overlook the findings that the peripheral application of inflammatory stimuli can lead to the activation of innate immunity, thereby exacerbating cerebral β-amyloidosis and ultimately contributing to cognitive decline (Netea et al., 2020). Thus, while our adjusted findings suggest a protective effect of certain infections in relation to dementia risk, they do not unequivocally support a generalized protective role against dementia. These results may be influenced by other factors, such as the balance between innate and adaptive immune responses, the specific nature of infections, and the overall immune health of individuals. In this regard, our study adds to the body of evidence on the relationship between infection and dementia, highlighting the importance of considering both innate and adaptive immune responses. Moreover, a previous study that found no significant association between VZV infection and the incidence of dementia observed that antiviral treatment was associated with a lower risk of dementia in secondary analysis (Zhu et al., 2024). This suggests that the association between VZV infection and dementia may be obscured by antiviral treatment, potentially through mechanisms such as reducing the risk of stroke, a known risk factor for dementia, or mitigating the subclinical activity of the virus. However, our study lacked sufficient data on the use of antiviral medication, making it challenging to assess the impact of antiviral treatment on the risk of developing dementia. Therefore, further research considering the treatment status of infections is warranted. Additionally, potential sampling bias may arise from the inclusion of healthy individuals who underwent thorough health screenings to confirm infections but had a lower risk of dementia. Furthermore, the possibility of diagnostic inaccuracies in long-term cohort studies adds to the complexity of these interpretations.

It is pertinent to consider that after adjusting for key dementia risk factors, such as diabetes mellitus, hypertension, dyslipidemia, and depression, the initially observed higher risk of dementia in the infection group either reduced or suggested a possible protective effect. This change in risk perception upon covariate control underscores the significance of these factors in the infection-dementia association. This suggests the possibility of confounding effects in the unadjusted models and that factors other than infections alone might have a more direct influence on dementia risk. This aspect adds another layer to our understanding, serving as a reminder of the complexities involved in fully discerning the true impact of infection on dementia risk. In this study, we performed a stepwise selection to fit the Cox regression model and found that depression emerged as the most influential modifiable variable on dementia risk, followed by DM. Therefore, among various covariates, adjusting for depression and DM significantly contributed to the observed reduction in risk post-adjustment. The relationship between depression and dementia has been extensively studied, highlighting depression as a significant risk factor for dementia, including AD (Piras et al., 2021). Research indicates that individuals with both depression and DM have an even higher risk of developing dementia. For example, studies have shown that patients with both conditions have a 100% increased risk of dementia compared to those with DM alone (Katon et al., 2015). This underscores the importance of considering both depression and DM in understanding and mitigating dementia risk. Further research is needed to explore the impact of infections on dementia, particularly in the context of coexisting depression and DM.

Third, we observed a consistent trend in both the CP and SCD groups when examining the relationship between the number of infectious agents and dementia onset. An increasing number of infectious agents are correlated with rising IRs of dementia. This trend was more pronounced in the CP group in the unadjusted models, indicating a potential increase in the risk of dementia with a greater number of infections. However, this trend did not persist in the adjusted models for either group, underscoring the need to consider confounding factors. Although our adjusted models suggest a protective role of infections, given the lack of strong evidence of infection for a protective effect in the adjusted models, the trends observed in the IR and unadjusted models cannot be entirely disregarded. While these findings should be interpreted with caution, our results contribute to the broader narrative of the polymicrobial causality hypothesis in AD, which posits that the cumulative infectious burden may be more relevant to AD onset than individual infections (Vigasova et al., 2021). Nonetheless, considering the discrepancy between the trend of increasing dementia risk with the number of infectious agents and the adjusted results, it is necessary to thoroughly examine the complexity of delineating the true impact of infection on dementia risk. In a previous study that followed 260,000 Finnish adults for 15 years in the primary cohort and 485,000 UK Biobank participants for 8 years in the replication cohort, the risk of developing dementia was examined not only based on the number of infections but also considering the invasiveness and severity of infections (Sipilä et al., 2021). Additionally, the impact of the chronicity of infections was assessed, particularly considering the persistence of herpes viruses in the body after the initial infection. While a dose-response relationship was observed between multiple episodes of infection and the risk of developing dementia, there were no significant differences in dementia risk based on the type, severity, or chronicity of the infections. In line with this, we observed no significant difference in the risk of dementia development based on the type of infectious agent, consistent across both the CP and SCD groups. This suggests that the type of infectious agent does not play a distinct role in influencing dementia risk among these groups. However, factors that might influence dementia risk, such as the severity or chronicity of the infectious agents, were not considered in this study. Moreover, the impact of the duration of the disease or the recurrence of diseases caused by the same infectious agent on dementia risk cannot be ignored. Chronic or recurrent infections might have a different impact on dementia risk compared to single, acute infections. Certain infections, like HSV and *H. pylori*, can persist for a long time or reactivate periodically, potentially contributing to chronic inflammation and continuous immune activation, which might influence cognitive decline. Weighting all infections equally might obscure the varying impacts of different pathogens. Future research could propose differential weighting based on factors like pathogenicity, chronicity, and severity. Lastly, future studies should aim to further unravel these intricate relationships by exploring other contributing factors to dementia risk and considering the cumulative effects of multiple infections within a broader epidemiological context. This could be due to biases, such as healthier individuals being more likely to have a history of infections due to greater medical surveillance, or confounding factors not fully accounted for.

Furthermore, when exploring the specific interplay between HSV and VZV, our study found that although these viruses are known for their potential to induce synergistic effects in brain inflammation and immune responses, particularly with VZV increasing Aβ burden and potentially activating latent HSV (Bubak et al., 2020; Cairns et al., 2022), the sequence of these infections did not significantly affect the risk of dementia onset. This finding emphasizes the need for more controlled clinical studies to reassess evidence from previous translational studies, thus contributing to a deeper understanding of the specific roles of these infections in dementia risk.

Additionally, our findings on the correlation between the number of infectious agents and onset of dementia are further accentuated when differentiating between AD and VaD. In both the CP and SCD groups, the risk of dementia onset, based on infection status and number of infectious agents, was more pronounced in patients with AD than in those with VaD in an unadjusted model. One proposed mechanism for this disparity is the inflammatory response triggered by infection. It has been posited that inflammation leads to the accumulation of Aβ, which in turn promotes AD progression by causing neuronal damage and death in the brain (Kinney et al., 2018). While inflammation can adversely affect blood vessels, leading to reduced blood flow and potentially setting the stage for VaD (Wang et al., 2020), it emerges as a pivotal mechanism in the initiation and progression of AD (Marchesi, 2011). Thus, chronic inflammation resulting from infections appears to have a more profound impact on the onset and progression of AD than VaD. This assertion aligns with our observations that the influence of infection was distinctly more marked in AD than in VaD. Furthermore, the lower prevalence of VaD relative to that of AD in our study cohort could have shaped these findings. However, a previous study observed the strongest association between infection and dementia incidence in cases of vascular dementia, suggesting that systemic inflammation caused by infections can lead to blood-brain barrier dysfunction, resulting in neuroinflammation, neuron loss, and ischemic damage (Sipilä et al., 2021). Thus, future research should focus on understanding the mechanisms by which infectious agents contribute to dementia, which would allow for a balanced selection of infectious agents for study and the assessment of their impact on various subtypes of dementia.

It is important to note several limitations of our study. Both SCD and the number of infections are established risk factors for dementia. However, this study does not definitively reveal whether infections cause dementia, worsen existing SCD, or both. Additionally, individuals with SCD or early dementia might be more likely to have multiple infections due to associated health conditions or lifestyle factors, making it challenging to isolate the true causal pathway. Future research should include stratified analyses and mediational analysis to determine the specific interactions and causal relationships between SCD, infections, and dementia risk. Additionally, the evaluation period for infection in NSPTA participants ranged from 2 to 10 years, and data on infection status during childhood, adolescence, and early adulthood were lacking. This gap indicates that the impact of past infections may not have been fully captured in assessing dementia risk. Future research would benefit from long-term prospective studies with extended observation periods to better understand the influence of these early life stages. Furthermore, participants in this study were enrolled at age 66 years and followed up until 2017, allowing us to examine dementia risk only up to the age of 76 years. Consequently, we lack data on dementia risk beyond this age, a period during which the incidence of dementia significantly increases (Alzheimer’s Association, 2019). Additional studies are needed to explore the risk of dementia onset beyond the age of 76. In addition, in this study, we used the KDSQ-P, a self-report scale, to assess the severity of SCD. According to this scale, scores of 4 or higher are considered clinically significant for cognitive decline. Among the SCD group, 89.1% of participants scored between 4 and 6. Given that most participants are distributed in the marginal score range and those diagnosed with dementia were excluded, it is believed that the severity of subjective cognitive decline does not compromise the reliability of the scale. While this study focused on analyzing the SCD group due to its clinical characteristics, it would be clinically meaningful to conduct similar analyses on groups exhibiting objective cognitive decline in future research.

Lastly, as this is an epidemiological investigation into the potential risk of infection on dementia onset, further research should focus on elucidating the underlying biological mechanisms. This includes factors such as Aβ deposition, *APOE* ε4 carrier status, brain atrophy severity, and inflammatory markers.

In summary, our study reaffirms that SCD acts as a consistent risk factor for dementia onset, a finding that aligns with existing literature. This relationship holds regardless of the presence or number of infections, emphasizing independent significance of SCD in the risk of dementia. We did not observe a meaningful interaction between infection status and SCD in influencing dementia risk, suggesting that role of SCD as a risk factor remains stable, irrespective of these factors. Our findings paint a more complex picture of the impact of infection on dementia. Initially, infections appeared to increase the risk of dementia, particularly in the CP group. However, this association disappeared after adjusting for key dementia risk factors, indicating that the direct impact of infection on dementia is nuanced and interwoven with a range of health conditions. While in some instances infections showed a potential protective effect, this aspect requires cautious interpretation and further investigation, underscoring the complexity of the relationship between infections and dementia. Overall, our findings emphasize that, while SCD is a steadfast risk factor for dementia, the role of infections is layered, subject to various influences, and requires more comprehensive exploration to fully understand their impact on dementia development.

## Data Availability

Publicly available datasets were analyzed in this study. This data can be found here: https://nhiss.nhis.or.kr/bd/ ab/bdaba000eng.do;jsessionid=BsvfdnoXck1szAGGuctHvtq0YXq QGlSlPZY1Usj4NrClau1WzXadfDntlLQaWfPE.primrose2_servlet_ engine10.
