# Supplementary material for: Impact of multiple infections on risk of incident dementia according to subjective cognitive decline status: a nationwide population-based cohort study
Source: Front Aging Neurosci. 2024 Sep 2;16:1410185. doi: 10.3389/fnagi.2024.1410185 (PMC11402823; doi:10.3389/fnagi.2024.1410185)
Supplement: Supplementary file 1 [file Table_1.pdf]

Supplementary Material

Impact of multiple infections on risk of incident dementia according to subjective cognitive decline status: A nationwide population-based cohort study

Jung-Won Lee[1], Mina Kim[2], Hoseob Kim[2], Sung-Hwan Kim[3], Yoo Hyun Um[4], Sheng-Min Wang[3], Hyun Kook Lim[3,5], Chang Uk Lee[1], and Dong Woo Kang[1]*

[1]Department of Psychiatry, Seoul St. Mary's Hospital, College of Medicine, The Catholic University of Korea, Seoul, Republic of Korea

[2]Department of Data Science, Hanmi Pharm. Co., Ltd, Seoul, Republic of Korea

[3]Department of Psychiatry, Yeouido St. Mary's Hospital, College of Medicine, The Catholic University of Korea, Seoul, Republic of Korea

[4]Department of Psychiatry, St. Vincent's Hospital, College of Medicine, The Catholic University of Korea, Suwon, Republic of Korea

[5]Research Institute, NEUROPHET Inc., Seoul, Republic of Korea

Running head: Infections, Cognitive Decline & Dementia

***Correspondence:** Dong Woo Kang, MD, PhD

Department of Psychiatry, Seoul St. Mary's Hospital, College of Medicine, The Catholic University of Korea, 222, Banpo-daero, Seocho-gu, Seoul, 06591, Republic of Korea

Tel: +82-2-2258-6084, Fax: +82-2-594-3870, E-mail: kato7@hanmail.net

Supplementary Methods and materials

1. Definition of demographic characteristics and medical history

The presence of diabetes mellitus was defined according to the presence of ≥1 claim per year under ICD-10 codes E10-14 and ≥1 claim per year for the prescription of anti-diabetic medication (ATC codes: A10A, A10B, A10X), if this condition was met once during hospitalization or on two or more occasions in outpatient settings. The presence of hypertension was defined according to the presence of ≥1 claim per year under ICD-10 codes I10-15 and ≥1 claim per year for the prescription of antihypertensive agents (ATC codes: C03, C07, C08, C09), if this condition was met once during hospitalization or on two or more occasions in outpatient settings. The presence of ischemic heart disease was defined according to the presence of ≥1 claim per year under ICD-10 codes I20-25. The presence of dyslipidemia was defined according to the presence of ≥1 claim per year under ICD-10 code E78 and ≥1 claim per year for the prescription of a lipid-lowering agent (ATC code: C10). The presence of depression was defined according to the presence of ≥1 claim per year under ICD-10 codes F32x or F33x and ≥1 claim per year for the prescription of antidepressant (ATC code: N06).

Supplementary Table S1. Sum of KDSQ-P score of study population

	**CP (n=825,405)**	**SCD (n=275,135)**	***P***
**Sum of KDSQ-P score [n(%)]**			<.0001
0	825,405(100)	0(0)	
1	0(0)	0(0)	
2	0(0)	0(0)	
3	0(0)	0(0)	
4	0(0)	106,242(38.6)	
5	0(0)	119,435(43.4)	
6	0(0)	19,554(7.1)	
7	0(0)	11,007(4)	
8	0(0)	7,365(2.7)	
9	0(0)	4,305(1.6)	
10	0(0)	7,227(2.6)	

CP, cognitively preserved older adults; SCD, subjective cognitive decline; KDSQ-P, Prescreening Korean Dementia Screening Questionnaire; *p* value by x^2 test.

Supplementary Table S2. Helicobacter pylori Eradication Regimens

Type	*H. pylori* eradication regimens
1	PPI + clarithromycin + amoxicillin
2	PPI + clarithromycin + metronidazole
3	PPI + amoxicillin + metronidazole
4	PPI + tetracyclin + amoxicillin
5	PPI + tetracyclin + metronidazole
6	PPI + clarithromycin + tetracyclin
7	PPI + bismuth + tetracyclin + metronidazole
8	PPI + bismuth + amoxicillin + metronidazole
9	PPI + levofloxacin + amoxicillin
10	PPI + bismuth + tetracyclin + levofloxacin
11	PPI + bismuth + amoxicillin + levofloxacin
12	H2RA + clarithromycin + amoxicillin
13	H2RA + clarithromycin + metronidazole
14	H2RA + amoxicillin + metronidazole
15	H2RA + tetracyclin + amoxicillin
16	H2RA + tetracyclin + metronidazole
17	H2RA + clarithromycin + tetracyclin
18	H2RA + bismuth + tetracyclin + metronidazole
19	H2RA + bismuth + amoxicillin + metronidazole
20	H2RA + levofloxacin + amoxicillin
21	H2RA + bismuth + tetracyclin + levofloxacin
22	H2RA + bismuth + amoxicillin + levofloxacin
23	PPI + clarithromycin + bismuth
24	PPI + amoxicillin + bismuth
25	PPI + metronidazole + bismuth
26	PPI + tetracyclin + bismuth
27	H2RA + clarithromycin + bismuth
28	H2RA + amoxicillin + bismuth
29	H2RA + metronidazole + bismuth
30	H2RA + tetracyclin + bismuth

NOTE. These drug combinations were prescribed within the same prescription order and the duration of therapy was between 7 and 14 days.
PPI, proton pump inhibitor

Supplementary Table S3. Risk of incident dementia according to the infection stratified by the number of infectious agents across cognitive status

(A) Cognitively preserved older adults

Infection History	Number	Overall dementia	Duration (Person years)	IR per 1000	Unadjusted HR (95% CI) (Model 1)			Adjusted HR (95% CI) (Model 2)			Adjusted HR (95% CI) (Model 3)		
					Hazard Ratio	Low	High	Hazard Ratio	Low	High	Hazard Ratio	Low	High
**Non-infection**	512,026	20,261	2,851,925.38	7.1		1(ref.)			1(ref.)			1(ref.)	
**Single infection**	233,732	8,310	1,197,091.41	6.9	**1.032**	**1.006**	**1.059**	0.987	0.962	1.013	0.919	0.887	0.951
H. pylori	53,273	1,719	271,458.16	6.3	0.944	0.899	0.992	0.937	0.891	0.984	0.873	0.819	0.931
HSV	75,144	2,896	387,173.21	7.5	**1.107**	**1.065**	**1.151**	**1.05**	**1.01**	**1.092**	0.966	0.916	1.019
VZV	94,558	3,329	484,354.15	6.9	1.022	0.985	1.06	0.965	0.93	1.001	0.903	0.859	0.949
HPV	10,757	366	54,085.89	6.8	1.017	0.917	1.128	0.986	0.889	1.093	0.965	0.844	1.104
**Dual infection**	71,213	2,447	343,275.51	7.1	**1.099**	**1.053**	**1.146**	1.012	0.971	1.056	0.881	0.831	0.933
H. pylori + HSV	9,225	302	44,183.40	6.8	1.058	0.944	1.185	0.994	0.887	1.113	0.859	0.738	1.000
H. pylori + VZV	11,051	367	52,704.91	7.0	1.081	0.975	1.198	1.012	0.913	1.122	0.936	0.819	1.069
H. pylori + HPV	1,406	39	6,592.33	5.9	0.926	0.676	1.268	0.899	0.656	1.23	0.704	0.459	1.08
HSV + VZV*	17,131	605	83,301.92	7.3	**1.115**	**1.028**	**1.209**	1.019	0.939	1.105	0.826	0.736	0.928
VZV + HSV[†]	27,107	978	131,242.51	7.5	**1.144**	**1.072**	**1.219**	1.046	0.98	1.115	0.905	0.827	0.99
VZV + HSV[‡]	44,238	1,583	214,544.44	7.4	**1.133**	**1.076**	**1.192**	1.035	0.983	1.09	0.874	0.813	0.94
HSV + HPV	2,620	74	12,448.10	5.9	0.924	0.736	1.161	0.848	0.675	1.066	0.816	0.607	1.097
VZV +HPV	2,673	82	12,802.34	6.4	0.993	0.799	1.233	0.908	0.731	1.128	0.992	0.757	1.300
**Triple infection**	8,152	294	37,479.16	7.8	**1.243**	**1.108**	**1.395**	**1.133**	**1.01**	**1.271**	0.934	0.797	1.095

H. pylori + HSV + VZV*	2,191	67	10,166.97	6.6	1.04	0.818	1.322	0.946	0.744	1.203	0.815	0.59	1.126
H. pylori + VZV + HSV†	3,428	141	15,717.08	9.0	**1.424**	**1.207**	**1.681**	**1.315**	**1.114**	**1.551**	1.087	0.869	1.361
H. pylori + VZV + HSV‡	5,619	208	25,884.04	8.0	**1.272**	**1.109**	**1.458**	**1.168**	**1.019**	**1.339**	0.981	0.816	1.18
H. pylori + HSV + HPV	378	7	1,738.14	4.0	0.642	0.306	1.345	0.595	0.284	1.249	0.517	0.194	1.378
H. pylori + VZV + HPV	374	13	1,658.44	7.8	1.27	0.737	2.187	1.165	0.676	2.006	1.141	0.571	2.283
HSV + VZV + HPV*	1,781	66	8,198.54	8.1	1.274	1	1.622	1.128	0.886	1.437	0.831	0.577	1.197
VZV + HSV + HPV†	748	27	3,443.80	7.8	1.243	0.853	1.814	1.099	0.754	1.603	0.758	0.42	1.369
VZV + HSV + HPV‡	1,033	39	4,754.74	8.2	1.296	0.946	1.774	1.149	0.839	1.574	0.883	0.556	1.403
**Quadruple infection**	282	6	1241.51	4.8	0.781	0.351	1.738	0.683	0.307	1.519	0.328	0.082	1.310
H. pyroli + HSV + VZV + HPV*	116	2	522.08	3.8	0.617	0.155	2.459	0.558	0.14	2.233	0.392	0.055	2.780
H. pyroli + VZV + HSV + HPV†	166	4	719.43	5.6	0.903	0.339	2.405	0.768	0.288	2.046	0.283	0.04	1.998
H. pyroli + HSV + VZV + HPV‡	282	6	1241.51	4.8	0.781	0.351	1.738	0.683	0.307	1.519	0.328	0.082	1.310

**Infection History**	**Number**	**Alzheimer's disease**	**Duration (Person years)**	**IR per 1000**	**Unadjusted HR (95% CI) (Model 1)**			**Adjusted HR (95% CI) (Model 2)**			**Adjusted HR (95% CI) (Model 3)**		
					**Hazard Ratio**	**Low**	**High**	**Hazard Ratio**	**Low**	**High**	**Hazard Ratio**	**Low**	**High**
**Non-infection**	512,026	19,575	2,851,925.38	6.9		1(ref.)			1(ref.)			1(ref.)	
**Single infection**	233,732	8,037	1,197,091.41	6.7	**1.035**	**1.008**	**1.062**	0.989	0.963	1.015	0.92	0.888	0.953
H. pylori	53,273	1,653	271,458.16	6.1	0.942	0.896	0.99	0.934	0.888	0.982	0.874	0.819	0.932
HSV	75,144	2,816	387,173.21	7.3	**1.116**	**1.072**	**1.161**	**1.056**	**1.015**	**1.099**	0.971	0.92	1.025
VZV	94,558	3,210	484,354.15	6.6	1.022	0.984	1.061	0.962	0.927	0.999	0.901	0.856	0.948
HPV	10,757	358	54,085.89	6.6	1.032	0.929	1.145	0.999	0.9	1.11	0.983	0.859	1.126

**Dual infection**	71,213	2,396	343,275.51	7.0	**1.117**	**1.07**	**1.165**	1.026	0.983	1.071	0.892	0.842	0.946
H. pylori + HSV	9,225	293	44,183.40	6.6	1.065	0.949	1.196	0.999	0.89	1.122	0.86	0.736	1.004
H. pylori + VZV	11,051	362	52,704.91	6.9	1.107	0.997	1.228	1.035	0.932	1.148	0.958	0.837	1.096
H. pylori + HPV	1,406	38	6,592.33	5.8	0.937	0.682	1.288	0.91	0.662	1.251	0.733	0.477	1.124
HSV + VZV*	17,131	588	83,301.92	7.1	**1.125**	**1.036**	**1.221**	1.024	0.943	1.112	0.832	0.739	0.936
VZV + HSV†	27,107	960	131,242.51	7.3	**1.165**	**1.092**	**1.243**	1.062	0.995	1.133	0.918	0.838	1.006
VZV + HSV‡	44,238	1,548	214,544.44	7.2	**1.149**	**1.091**	**1.211**	1.047	0.994	1.103	0.884	0.822	0.952
HSV + HPV	2,620	73	12,448.10	5.9	0.947	0.752	1.192	0.867	0.689	1.091	0.826	0.612	1.114
VZV +HPV	2,673	82	12,802.34	6.4	1.03	0.829	1.28	0.941	0.757	1.169	1.028	0.785	1.346
**Triple infection**	8,152	287	37,479.16	7.7	**1.261**	**1.122**	**1.417**	**1.146**	**1.02**	**1.288**	0.95	0.809	1.115
H. pylori + HSV + VZV*	2,191	66	10,166.97	6.5	1.064	0.836	1.355	0.965	0.758	1.229	0.844	0.611	1.166
H. pylori + VZV + HSV†	3,428	137	15,717.08	8.7	**1.438**	**1.216**	**1.701**	**1.324**	**1.119**	**1.567**	1.098	0.875	1.379
H. pylori + VZV + HSV‡	5,619	203	25,884.04	7.8	**1.29**	**1.123**	**1.481**	**1.181**	**1.029**	**1.357**	0.999	0.829	1.204
H. pylori + HSV + HPV	378	6	1,738.14	3.5	0.575	0.259	1.275	0.529	0.238	1.178	0.537	0.201	1.13
H. pylori + VZV + HPV	374	13	1,658.44	7.8	1.321	0.767	2.275	1.208	0.701	2.081	1.189	0.594	2.378
HSV + VZV + HPV*	1,781	65	8,198.54	7.9	1.292	0.886	1.885	1.138	0.78	1.66	0.785	0.435	1.419
VZV + HSV + HPV†	748	27	3,443.80	7.8	1.312	0.954	1.803	1.159	0.843	1.594	0.863	0.536	1.39
VZV + HSV + HPV‡	1,033	38	4,754.74	8.0	**1.304**	**1.022**	**1.663**	1.15	0.902	1.468	0.831	0.573	1.204
**Quadruple infection**	282	6	1241.51	4.8	0.812	0.365	1.807	0.707	0.318	1.574	0.339	0.085	1.356
H. pyroli + HSV + VZV + HPV*	116	2	522.08	3.8	0.939	0.352	2.502	0.795	0.298	2.118	0.293	0.041	2.069
H. pyroli + VZV + HSV + HPV†	166	4	719.43	5.6	0.812	0.365	1.807	0.707	0.318	1.574	0.339	0.085	1.356

H. pyroli + HSV + VZV + HPV‡	282	6	1241.51	4.8	0.641	0.161	2.555	0.579	0.145	2.316	0.405	0.057	2.878

**Infection History**	**Number**	**Vascular dementia**	**Duration (Person years)**	**IR per 1000**	**Unadjusted HR (95% CI) (Model 1)**			**Adjusted HR (95% CI) (Model 2)**			**Adjusted HR (95% CI) (Model 3)**		
					**Hazard Ratio**	**Low**	**High**	**Hazard Ratio**	**Low**	**High**	**Hazard Ratio**	**Low**	**High**
**Non-infection**	512,026	555	2,851,925.38	0.2		1(ref.)			1(ref.)			1(ref.)	
**Single infection**	233,732	237	1,197,091.41	0.2	1.012	0.869	1.179	1.012	0.868	1.179	0.929	0.761	1.134
H. pylori	53,273	61	271,458.16	0.2	1.149	0.882	1.497	1.125	0.863	1.467	0.968	0.689	1.362
HSV	75,144	68	387,173.21	0.2	0.898	0.698	1.156	0.905	0.703	1.165	0.858	0.615	1.197
VZV	94,558	101	484,354.15	0.2	1.066	0.862	1.318	1.072	0.866	1.326	1.002	0.757	1.326
HPV	10,757	7	54,085.89	0.1	0.661	0.314	1.393	0.655	0.311	1.381	0.573	0.214	1.537
**Dual infection**	71,213	45	343,275.51	0.1	0.667	0.492	0.904	0.667	0.492	0.905	0.63	0.424	0.935
H. pylori + HSV	9,225	9	44,183.40	0.2	1.036	0.536	2.002	1.022	0.529	1.976	0.997	0.444	2.238
H. pylori + VZV	11,051	3	52,704.91	0.1	0.289	0.093	0.9	0.285	0.092	0.888	0.277	0.069	1.114
H. pylori + HPV	1,406	1	6,592.33	0.2	0.769	0.108	5.472	0.747	0.105	5.316	0	0	-
HSV + VZV*	17,131	14	83,301.92	0.2	0.856	0.503	1.455	0.865	0.508	1.471	0.806	0.399	1.629
VZV + HSV†	27,107	17	131,242.51	0.1	0.659	0.407	1.068	0.664	0.41	1.077	0.641	0.341	1.206
VZV + HSV‡	44,238	31	214,544.44	0.1	0.735	0.512	1.056	0.742	0.516	1.066	0.706	0.438	1.137
HSV + HPV	2,620	1	12,448.10	0.1	0.408	0.057	2.902	0.406	0.057	2.885	0.641	0.09	4.564
VZV +HPV	2,673	0	12,802.34	0.0	0	0	-	0	0		0	0	-
**Triple infection**	8,152	6	37,479.16	0.2	0.811	0.363	1.815	0.808	0.361	1.807	0.603	0.193	1.884
H. pylori + HSV + VZV*	2,191	1	10,166.97	0.1	0.499	0.07	3.551	0.498	0.07	3.544	0	0	-

H. pylori + VZV + HSV†	3,428	4	15,717.08	0.3	1.289	0.482	3.449	1.284	0.48	3.434	0.929	0.231	3.736
H. pylori + VZV + HSV‡	5,619	5	25,884.04	0.2	0.979	0.406	2.363	0.976	0.404	2.355	0.575	0.143	2.312
H. pylori + HSV + HPV	378	0	1,738.14	0.0	0	0	-	0	0	-	0	0	-
H. pylori + VZV + HPV	374	0	1,658.44	0.0	0	0	-	0	0		0	0	-
HSV + VZV + HPV*	1,781	1	8,198.54	0.1	0	0	3.28E+252	0	0	2.98E+252	0	0	-
VZV + HSV + HPV†	748	1	3,443.80	0.3	1.066	0.15	7.580	1.06	0.149	7.543	1.715	0.241	12.221
VZV + HSV + HPV‡	1,033	0	4,754.74	0.0	0.618	0.087	4.394	0.615	0.087	4.377	0.991	0.139	7.063
**Quadruple infection**	282	0	1241.51	0.0	0	0	-	0	0	-	0	0	-
H. pyroli + HSV + VZV + HPV*	116	0	522.08	0.0	0	0	-	0	0	-	0	0	-
H. pyroli + VZV + HSV + HPV†	166	0	719.43	0.0	0	0	-	0	0	-	0	0	-
H. pyroli + HSV + VZV + HPV‡	282	0	1241.51	0.0	0	0	-	0	0	-	0	0	-

(B) Participants with subjective cognitive decline

Infection History	Number	Overall dementia	Duration (Person years)	IR per 1000	Unadjusted HR (95% CI) (Model 1)			Adjusted HR (95% CI) (Model 2)			Adjusted HR (95% CI) (Model 3)		
					Hazard Ratio	Low	High	Hazard Ratio	Low	High	Hazard Ratio	Low	High
**Non-infection**	169,241	15,690	967,844.90	16.2		1(ref.)			1(ref.)			1(ref.)	
**Single infection**	78,116	6,402	407,900.80	15.7	0.991	0.963	1.021	0.961	0.933	0.99	0.89	0.856	0.925
H. pylori	17,890	1,397	92,842.64	15.0	0.952	0.901	1.005	0.933	0.883	0.986	0.901	0.841	0.966
HSV	25,093	2,163	131,639.90	16.4	1.036	0.991	1.084	1.004	0.96	1.051	0.897	0.843	0.955
VZV	31,444	2,549	164,394.48	15.5	0.98	0.939	1.022	0.944	0.905	0.985	0.88	0.831	0.931

HPV	3,689	293	19,023.78	15.4	0.977	0.87	1.097	0.944	0.841	1.06	0.864	0.74	1.01
**Dual infection**	24,546	1,904	119,804.96	15.9	1.019	0.971	1.069	0.964	0.919	1.012	0.852	0.799	0.908
H. pylori + HSV	3,163	247	15,316.09	16.1	1.036	0.914	1.175	0.991	0.874	1.124	0.82	0.692	0.971
H. pylori + VZV	3,843	295	18,615.64	15.8	1.018	0.907	1.142	0.965	0.86	1.083	0.875	0.754	1.016
H. pylori + HPV	473	36	2,228.19	16.2	1.045	0.753	1.449	1.003	0.723	1.391	1.073	0.73	1.577
HSV + VZV*	5,882	782	28,830.68	16.7	1.07	0.977	1.172	1.018	0.929	1.114	0.875	0.773	0.991
VZV + HSV†	9,289	706	45,645.09	15.5	0.99	0.918	1.068	0.931	0.863	1.004	0.822	0.742	0.91
VZV + HSV‡	15,171	1,188	74,475.77	16.0	1.021	0.963	1.083	0.964	0.909	1.023	0.842	0.777	0.913
HSV + HPV	941	79	4,568.09	17.3	1.107	0.888	1.381	1.04	0.834	1.298	0.995	0.751	1.317
VZV +HPV	955	59	4,601.18	12.8	0.827	0.64	1.068	0.779	0.604	1.007	0.762	0.554	1.049
**Triple infection**	3,100	245	14,163.96	17.3	1.122	0.989	1.273	1.048	0.923	1.189	0.857	0.724	1.014
H. pylori + HSV + VZV*	864	65	4,042.04	16.1	1.041	0.816	1.328	0.984	0.771	1.255	0.746	0.532	1.045
H. pylori + VZV + HSV†	1,217	101	5,526.80	18.3	1.185	0.974	1.441	1.104	0.908	1.342	0.941	0.728	1.215
H. pylori + VZV + HSV‡	2,081	166	9,568.84	17.3	1.124	0.964	1.31	1.053	0.904	1.228	0.859	0.7	1.054
H. pylori + HSV + HPV	152	15	637.43	22.3	1.462	0.882	2.423	1.357	0.818	2.251	0.766	0.344	1.705
H. pylori + VZV + HPV	152	16	670.90	23.8	1.566	0.96	2.553	1.455	0.891	2.376	1.435	0.794	2.593
HSV + VZV + HPV*	715	48	3,250.79	14.8	0.958	0.722	1.272	0.886	0.667	1.176	0.752	0.519	1.09
VZV + HSV + HPV†	293	38	1,353.34	28.1	1.195	0.807	1.768	1.098	0.742	1.626	0.95	0.582	1.552
VZV + HSV + HPV‡	422	10	1,897.45	5.3	0.789	0.524	1.188	0.732	0.486	1.101	0.589	0.334	1.037
**Quadruple infection**	132	10	568.13	17.6	1.158	0.623	2.153	1.105	0.594	2.054	0.789	0.354	1.757
H. pyroli + HSV + VZV + HPV*	52	3	225.60	13.3	0.871	0.281	2.701	0.819	0.264	2.541	0.766	0.192	3.065

H. pyroli + VZV + HSV + HPV†	80	7	342.53	20.4	1.351	0.644	2.833	1.298	0.619	2.724	0.801	0.3	2.134
H. pyroli + HSV + VZV + HPV‡	132	10	568.13	17.6	1.158	0.623	2.153	1.105	0.594	2.054	0.789	0.354	1.757

**Infection History**	**Number**	**Alzheimer's disease**	**Duration (Person years)**	**IR per 1000**	**Unadjusted HR (95% CI) (Model 1)**			**Adjusted HR (95% CI) (Model 2)**			**Adjusted HR (95% CI) (Model 3)**		
					**Hazard Ratio**	**Low**	**High**	**Hazard Ratio**	**Low**	**High**	**Hazard Ratio**	**Low**	**High**
**Non-infection**	169,241	15,181	967,844.90	15.7		1(ref.)			1(ref.)			1(ref.)	
**Single infection**	78,116	6,229	407,900.80	15.3	0.998	0.969	1.028	0.967	0.938	0.996	0.895	0.86	0.932
H. pylori	17,890	1,364	92,842.64	14.7	0.962	0.91	1.017	0.944	0.893	0.997	0.912	0.85	0.978
HSV	25,093	2,102	131,639.90	16.0	1.042	0.996	1.091	1.008	0.963	1.056	0.897	0.843	0.956
VZV	31,444	2,479	164,394.48	15.1	0.986	0.945	1.029	0.949	0.909	0.99	0.887	0.838	0.939
HPV	3,689	284	19,023.78	14.9	0.981	0.872	1.103	0.946	0.842	1.064	0.867	0.74	1.015
**Dual infection**	24,546	1,846	119,804.96	15.4	1.023	0.975	1.074	0.966	0.821	1.015	0.852	0.799	0.91
H. pylori + HSV	3,163	240	15,316.09	15.7	1.043	0.918	1.185	0.997	0.877	1.133	0.836	0.705	0.992
H. pylori + VZV	3,843	287	18,615.64	15.4	1.026	0.913	1.153	0.971	0.864	1.092	0.87	0.747	1.013
H. pylori + HPV	473	35	2,228.19	15.7	1.053	0.756	1.467	1.011	0.726	1.409	1.068	0.721	1.582
HSV + VZV*	5,882	469	28,830.68	16.3	1.079	0.984	1.182	1.023	0.933	1.122	0.886	0.781	1.005
VZV + HSV†	9,289	684	45,645.09	15.0	0.994	0.921	1.073	0.932	0.863	1.006	0.82	0.74	0.909
VZV + HSV‡	15,171	1,153	74,475.77	15.5	1.027	0.967	1.09	0.967	0.91	1.027	0.845	0.779	0.917
HSV + HPV	941	75	4,568.09	16.4	1.089	0.868	1.367	1.021	0.814	1.281	0.944	0.704	1.266
VZV +HPV	955	56	4,601.18	12.2	0.813	0.626	1.058	0.765	0.588	0.995	0.747	0.538	1.036
**Triple infection**	3,100	240	14,163.96	16.9	**1.14**	**1.003**	**1.295**	1.062	0.934	1.206	0.88	0.743	1.042

H. pylori + HSV + VZV*	864	64	4,042.04	15.8	1.063	0.931	1.358	1.002	0.784	1.281	0.772	0.551	1.082
H. pylori + VZV + HSV†	1,217	100	5,526.80	18.1	1.217	0.999	1.481	1.13	0.928	1.376	0.973	0.753	1.257
H. pylori + VZV + HSV‡	2,081	164	9,568.84	17.1	1.151	0.987	1.343	1.076	0.923	1.256	0.888	0.724	1.09
H. pylori + HSV + HPV	152	14	637.43	20.8	1.414	0.838	2.387	1.311	0.777	2.215	0.794	0.356	1.767
H. pylori + VZV + HPV	152	15	670.90	22.4	1.52	0.917	2.52	1.414	0.852	2.346	1.488	0.824	2.688
HSV + VZV + HPV*	715	47	3,250.79	14.5	0.973	0.731	1.296	0.897	0.673	1.194	0.748	0.513	1.092
VZV + HSV + HPV†	293	37	1,353.34	27.3	1.189	0.797	1.774	1.09	0.73	1.627	0.918	0.553	1.524
VZV + HSV + HPV‡	422	10	1,897.45	5.3	0.818	0.544	1.232	0.756	0.503	1.139	0.608	0.345	1.071
**Quadruple infection**	132	10	568.13	17.6	1.203	0.647	2.236	1.145	0.616	2.129	0.817	0.367	1.82
H. pyroli + HSV + VZV + HPV*	52	7	225.60	31.0	0.904	0.291	2.80E+00	0.848	0.273	2.63E+00	0.792	0.198	3.168
H. pyroli + VZV + HSV + HPV†	80	10	342.53	29.2	1.404	0.669	2.94E+00	1.348	0.642	2.83E+00	0.83	0.312	2.213
H. pyroli + HSV + VZV + HPV‡	132	10	568.13	17.6	1.203	0.647	2.236	1.145	0.616	2.129	0.817	0.367	1.82

Infection History	Number	Vascular dementia	Duration (Person years)	IR per 1000	Unadjusted HR (95% CI) (Model 1)			Adjusted HR (95% CI) (Model 2)			Adjusted HR (95% CI) (Model 3)		
					Hazard Ratio	Low	High	Hazard Ratio	Low	High	Hazard Ratio	Low	High
**Non-infection**	169,241	394	967,844.90	0.4		1(ref.)			1(ref.)			1(ref.)	
**Single infection**	78,116	143	407,900.80	0.4	0.835	0.69	1.012	0.842	0.695	1.02	0.767	0.594	0.99
H. pylori	17,890	26	92,842.64	0.3	0.666	0.448	0.991	0.643	0.432	0.957	0.647	0.4	1.047
HSV	25,093	50	131,639.90	0.4	0.907	0.676	1.217	0.928	0.691	1.247	0.965	0.662	1.407
VZV	31,444	60	164,394.48	0.4	0.87	0.663	1.141	0.891	0.678	1.169	0.689	0.465	1.022
HPV	3,689	7	19,023.78	0.4	0.872	0.416	1.842	0.86	0.407	1.816	0.794	0.295	2.135

**Dual infection**	24,546	49	119,804.96	0.4	0.955	0.709	1.286	0.975	0.724	1.314	0.928	0.631	1.363
H. pylori + HSV	3,163	6	15,316.09	0.4	0.912	0.407	2.043	0.906	0.404	2.03	0.441	0.11	1.777
H. pylori + VZV	3,843	7	18,615.64	0.4	0.876	0.415	1.85	0.874	0.414	1.847	1.289	0.607	2.739
H. pylori + HPV	473	1	2,228.19	0.4	1.038	0.146	7.386	0.995	0.14	7.085	1.479	0.207	10.553
HSV + VZV*	5,882	12	28,830.68	0.4	0.974	0.549	1.731	1.015	0.571	1.804	0.688	0.283	1.673
VZV + HSV†	9,289	18	45,645.09	0.4	0.922	0.575	1.48	0.957	0.596	1.536	0.932	0.507	1.712
VZV + HSV‡	15,171	30	74,475.77	0.4	0.943	0.65	1.367	0.979	0.675	1.421	0.839	0.503	1.399
HSV + HPV	941	2	4,568.09	0.4	1.022	0.255	4.102	1.021	0.254	4.098	1.558	0.387	6.276
VZV +HPV	955	3	4,601.18	0.7	1.512	0.486	4.71	1.526	0.49	4.755	1.518	0.377	6.114
**Triple infection**	3,100	5	14,163.96	0.4	0.809	0.335	1.956	0.818	0.338	1.977	0.237	0.033	1.694
H. pylori + HSV + VZV*	864	1	4,042.04	0.2	0.569	0.08	4.051	0.577	0.081	4.109	0	0	-
H. pylori + VZV + HSV†	1,217	1	5,526.80	0.2	0.415	0.058	2.951	0.422	0.059	3.003	0	0	2.19E+294
H. pylori + VZV + HSV‡	2,081	2	9,568.84	0.2	0.48	0.12	1.925	0.487	0.121	1.957	0	0	1.32E+222
H. pylori + HSV + HPV	152	1	637.43	1.5	3.372	0.474	24.005	3.305	0.464	23.532	0	0	-
H. pylori + VZV + HPV	152	1	670.90	1.5	3.391	0.476	24.14	3.267	0.459	23.259	0	0	-
HSV + VZV + HPV*	715	1	3,250.79	0.3	0.705	0.099	5.019	0.716	0.101	5.094	1.108	0.155	7.915
VZV + HSV + HPV†	293	0	1,353.34	0.0	1.703	0.239	12.122	1.717	0.241	12.221	2.5	0.35	17.858
VZV + HSV + HPV‡	422	1	1,897.45	0.5	0	0	2.65E+139	0	0	2.88E+139	0	0	-
**Quadruple infection**	132	0	568.13	0.0	0	0	7.94E+152	0	0	1.34E+154	0	0	-
H. pyroli + HSV + VZV + HPV*	52	0	225.60	0.0	0	0	-	0	0	-	0	0	-
H. pyroli + VZV + HSV + HPV†	80	0	342.53	0.0	0	0	-	0	0	-	0	0	-

H. pyroli + HSV + VZV + HPV‡	132	0	568.13	0.0	0	0	7.94E+152	0	0	1.34E+154	0	0	-

P-value by t-test for continuous variables and by x^2 test for categorical variables. CP, cognitively preserved older adults; SCD, subjective cognitive decline; KDSQ-P, Prescreening Korean Dementia Screening Questionnaire; H. pylori, Helicobacter pylori; HSV, herpes simplex virus; VZV, varicella zoster virus; HPV, human papillomavirus; SD, standard deviation; *, HSV infection followed by VZV infection; †, VZV infection followed by HSV infection; ‡, infection order not considered. Values with a statistically significant increase in HR are highlighted in bold.

Supplementary Table S4. Stepwise Cox Regression Analysis of Covariates Influencing Dementia Risk

(A) Univariate Analysis:

		**Overall survival**							
		**Univariate**				**Multivariate**			
			**95% CI**				**95% CI**		
		**HR**	**Low**	**High**	**P**	**HR**	**Low**	**High**	**P**
**Age**		1.156	1.152	1.161	<.0001	1.144	1.139	1.15	<.0001
**Sex**									
	Male		reference				reference		
	Female	1.13	1.111	1.149	<.0001	1.147	1.105	1.191	<.0001
**Diabetes mellitus**									
	No		reference		<.0001		reference		<.0001
	Yes	1.736	1.707	1.765		1.42	1.385	1.455	
**Dyslipidemia**									
	No		reference		<.0001		reference		0.0075
	Yes	1.421	1.395	1.448		0.963	0.937	0.99	
**Hypertension**									
	No		reference		<.0001		reference		<.0001
	Yes	1.528	1.5	1.555		1.19	1.158	1.221	
**Ischemic heart disease**									
	No		reference		<.0001		reference		<.0001
	Yes	1.506	1.479	1.534		1.136	1.106	1.166	
**Depression**									
	No		reference		<.0001		reference		<.0001
	Yes	2.555	2.511	2.599		2.302	2.247	2.359	

Each variable was individually included in the model to assess its independent effect on dementia risk. The univariate analysis results highlighted the significance of each variable.

(B) Stepwise Selection Results

Analysis of Maximum Likelihood Estimates							
**Parameter**		**DF**	**Parameter Estimate**	**Standard Error**	**Chi-Square**	**Pr > Chi-Square**	**Hazard Ratio**
**Age**		1	0.13488	0.00242	3112.72	<.0001	1.144
**Sex**	Female	1	0.13705	0.01906	51.7221	<.0001	1.147
	Male	0	0				
**Diabetes mellitus**	Diabetes mellitus	1	0.35032	0.01249	786.1319	<.0001	1.42
	No diabetes mellitus	0	0				
**Dyslipidemia**	Dyslipidemia	1	-0.03761	0.01407	7.1474	0.0075	0.963
	No dyslipidemia	0	0				
**Hypertension**	Hypertension	1	0.17356	0.01352	164.7238	<.0001	1.19
	No hypertension	0	0				
**Ischemic heart disease**	Ischemic heart disease	1	0.12711	0.01363	87.0255	<.0001	1.136
	No ischemic heart disease	0	0				
**Depression**	Depression	1	0.83391	0.01239	4531.59	<.0001	2.302
	No depression	0	0				

This method combines forward selection and backward elimination, with a significance level of 0.05 for entry and stay in the model. The final model, obtained through stepwise selection, includes the significant variables and their impact.

(C) Summary of Stepwise selection

	Effect			Number	Score	Wald	
**Step**	**Entered**	**Removed**	**DF**	**In**	**Chi-Square**	**Chi-Square**	**Pr > ChiSq**
**1**	Depression		1	1	7030.8837		<.0001
**2**	Age		1	2	3572.1871		<.0001
**3**	Diabetes mellitus		1	3	1252.0604		<.0001
**4**	Hypertension		1	4	226.9416		<.0001
**5**	Ischemic heart disease		1	5	79.292		<.0001
**6**	Sex		1	6	48.958		<.0001
**7**	Dyslipidemia		1	7	7.1476		0.0075

(D) Model fit statistics

Model Fit Statistics		
**Criterion**	**Without Covariates**	**With Covariates**
**-2 LOG L**	800931.29	789839.76
**AIC**	800931.29	789863.76
**SBC**	800931.29	789963.85

Akaike Information Criterion (AIC): Lower values indicate a better model. The AIC decreased from 800931.29 to 789839.76. Schwarz Bayesian Criterion (SBC): Similar to AIC but penalizes more for model complexity. The SBC decreased from 800931.29 to 789963.85.
